# What role for cellular metabolism in the control of hepatitis viruses?

**DOI:** 10.3389/fimmu.2022.1033314

**Published:** 2022-11-17

**Authors:** Olivier Diaz, Pierre-Olivier Vidalain, Christophe Ramière, Vincent Lotteau, Laure Perrin-Cocon

**Affiliations:** ^1^ CIRI, Centre International de Recherche en Infectiologie, Team VIRal Infection, Metabolism and Immunity, Univ Lyon, Inserm, U1111, Université Claude Bernard Lyon 1, CNRS, UMR5308, ENS de Lyon, Lyon, France; ^2^ Laboratoire de Virologie, Hôpital de la Croix-Rousse, Hospices Civils de Lyon, Lyon, France

**Keywords:** cellular metabolism, immunometabolism, hepatitis virus, hepatocyte, innate immunity, liver diseases, inflammation

## Abstract

Hepatitis B, C and D viruses (HBV, HCV, HDV, respectively) specifically infect human hepatocytes and often establish chronic viral infections of the liver, thus escaping antiviral immunity for years. Like other viruses, hepatitis viruses rely on the cellular machinery to meet their energy and metabolite requirements for replication. Although this was initially considered passive parasitism, studies have shown that hepatitis viruses actively rewire cellular metabolism through molecular interactions with specific enzymes such as glucokinase, the first rate-limiting enzyme of glycolysis. As part of research efforts in the field of immunometabolism, it has also been shown that metabolic changes induced by viruses could have a direct impact on the innate antiviral response. Conversely, detection of viral components by innate immunity receptors not only triggers the activation of the antiviral defense but also induces in-depth metabolic reprogramming that is essential to support immunological functions. Altogether, these complex triangular interactions between viral components, innate immunity and hepatocyte metabolism may explain why chronic hepatitis infections progressively lead to liver inflammation and progression to cirrhosis, fibrosis and hepatocellular carcinoma (HCC). In this manuscript, we first present a global overview of known connections between the innate antiviral response and cellular metabolism. We then report known molecular mechanisms by which hepatitis viruses interfere with cellular metabolism in hepatocytes and discuss potential consequences on the innate immune response. Finally, we present evidence that drugs targeting hepatocyte metabolism could be used as an innovative strategy not only to deprive viruses of key metabolites, but also to restore the innate antiviral response that is necessary to clear infection.

## Introduction

Studies in the immunometabolism field have identified important connections between the cell metabolic status and innate immunity functions. The detection of viral components by pattern recognition receptors (PRRs) induces intracellular signaling that results in the activation of antiviral defenses but also triggers in-depth metabolic reprogramming that is essential to support immunological functions. Conversely, chronic metabolic disorders such as obesity or non-alcoholic fatty liver disease (NAFLD) are characterized by impaired innate antiviral defenses and deleterious chronic inflammation. This led to the concept of immunometabolic pathologies that are associated with poor outcomes in viral infections such as SARS-CoV-2 (1). Therefore, innate immunity and metabolic pathways interact in a reciprocal manner. Besides, viruses which are intracellular parasites have developed diverse strategies to hijack the cellular machinery to fulfill their needs in energy and molecular blocks to replicate. Recent studies suggest that the manipulation of cellular metabolism by viruses is also an evolutionarily selected strategy to control the innate immune response of infected cells (2). Deciphering the mechanisms involved should provide key information to design innovative antiviral therapies targeting immunometabolic regulations.

The liver is a central organ in metabolic homeostasis, controlling levels of macronutrients such as glucose, lipids and cholesterol. This role is mainly devoted to hepatocytes, which are the primary epithelial cell population in the liver. The liver is also involved in the endocrine control of growth signaling pathways and supports the immune response through the secretion of acute phase proteins and cytokines. This organ is therefore a hub where metabolic and innate immune processes connect. Hepatitis viruses such as HBV, HCV and HDV infect specifically hepatocytes and often establish chronic viral infections of the liver, escaping viral immunity for years. These infections promote metabolic disorders associated with chronic inflammation that lead to fibrosis, cirrhosis and hepatocellular carcinoma (HCC). Metabolic reprogramming associated with immunological alterations are key drivers in disease progression, but interactions between these two components are still poorly understood. Here, we first summarize our current understanding of functional connections between metabolism and innate immunity pathways at the cellular level. We then present the impact of hepatitis viruses on hepatocyte metabolism, and then discuss potential consequences on innate immunity. Finally, we show that understanding immunometabolic interactions in hepatocytes opens perspectives in the development of dual-effect antiviral therapies that would simultaneously starve viruses for key metabolites and stimulate the innate antiviral response.

## Cross-talk between cellular metabolism and key signaling pathways of the innate antiviral response: An overview

**Figure 1:**
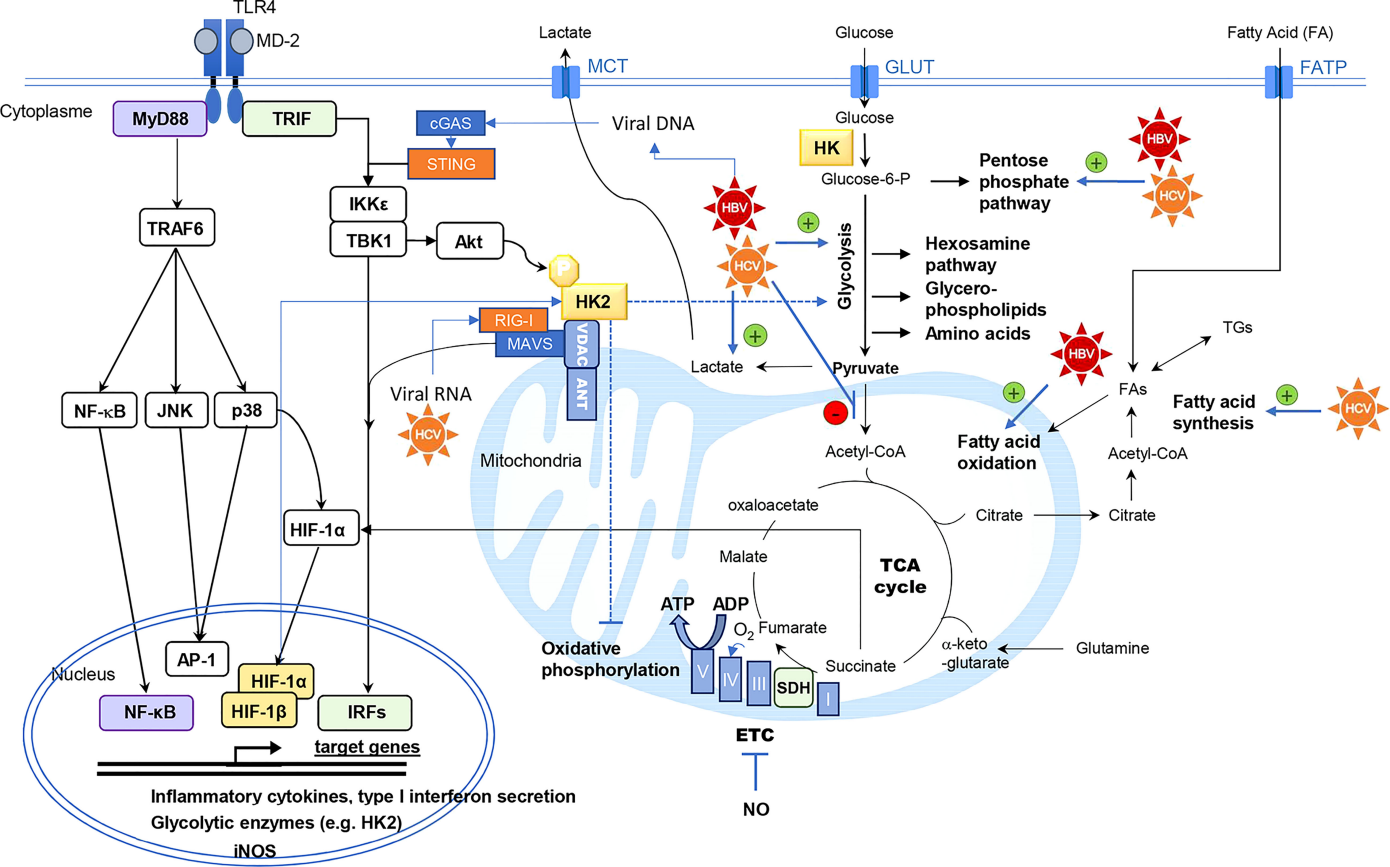
Interconnexion between innate immunity signaling pathways and central carbon metabolism of the cell. Hexokinase (HK) activity is the rate-limiting enzyme controlling glucose entry into glycolysis. Intermediary metabolites of glycolysis are precursors of anabolic pathways (pentose phosphate pathway, hexosamine pathway, glycerol-phospholipids and amino acids biosynthesis). Pyruvate is converted to acetyl-CoA into the mitochondria, fueling the tricarboxylic acid cycle (TCA) cycle. This cycle is coupled to oxidative phosphorylation by the succinate dehydrogenase (SDH) which is the complex II of the electron transfer chain (ETC). Under aerobic conditions, electron transport through the ETC (Complex I to V) generates ATP by oxidative phosphorylation. HCV and HBV have been described as modulators of the central carbon metabolism by different mechanisms, targeting glycolysis, lactate production, mitochondrial usage of pyruvate, fatty acid oxidation or synthesis. Thereby these viruses can rewire the flow of metabolites in these connected metabolic pathways. Viral RNA and DNA are detected by retinoic acid-inducible gene I (RIG-I) and cGAS respectively. RIG-I signaling is mediated by mitochondrial antiviral signaling protein (MAVS) polymerization at the mitochondrial membrane, triggering TANK-binding kinase 1 (TBK1)/ inhibitor of NF-κB kinase subunit epsilon (IKKε) activation. The stimulator of IFN genes protein (STING) is an essential signal transducer of cGAS and it also functions as an adapter in the sensing of RNA viruses via RIG-I. Infected cells also produce danger signals such as high mobility group box 1 (HMGB1) which is a ligand of Toll-like receptor (TLR) 4. TLR4 stimulation results in the activation of nuclear factor-κB (NFκB), c-Jun N-terminal kinase (JNK), p38-mitogen activated protein kinase (MAPK), inducing the secretion of pro-inflammatory cytokines, and of TBK1/IKKε inducing interferon response factors (IRFs)-dependent type I interferon secretion and phosphorylation of protein kinase B/Akt. Hexokinase-2 (HK2) phosphorylation at Thr473 by Akt promotes HK2 binding to mitochondrial voltage-dependent anion channel (VDAC), where it also interacts with MAVS, the signaling adaptor of RIG-I. Mitochondrial binding of HK2 is associated with enhanced glycolytic and reduced oxidative phosphorylation activities. In human monocyte-derived DCs, p38-MAPK activation results in hypoxia-induced factor (HIF)-1α accumulation, enhancing the expression of metabolic enzymes such as HK2, inducible nitric oxide synthase (iNOS) and pro-IL-1β. Nitric oxide (NO) radical produced by iNOS can inhibit the mitochondrial respiratory chain. When succinate accumulates in the cell, this metabolite inhibits prolyl-hydroxylase domain (PHD) enzymes that degrade HIF-1α thus favoring its accumulation. Adenine nucleotide translocase (ANT), fatty acid (FA), fatty acid transporter (FATP), glucose transporter (GLUT), monocarboxylic acid transporter (MCT), triglyceride (TG).

Cellular metabolism can greatly vary during activation of the innate immune response and it has been widely observed that reprogramming of cell metabolism can reallocate cellular resources for the achievement of specific immune functions (3). Indeed, cells are using carbohydrates, especially glucose that is degraded by glycolysis into pyruvate, to produce ATP and metabolic precursors for other pathways (**Figure 1**). Pyruvate is converted to acetyl-CoA into the mitochondria, fueling the tricarboxylic acid cycle (TCA) cycle, which is coupled to the respiratory chain and oxidative phosphorylation (OXPHOS) in the presence of oxygen, generating up to 36 molecules of ATP per molecule of glucose. Although glycolysis is producing only 2 molecules of ATP per molecule of glucose, it can be more rapidly engaged and increased to meet the energetic demand of activated proinflammatory immune cells. Furthermore, intermediate metabolites of glycolysis are fueling several anabolic pathways for the synthesis of lipids, carbohydrates, nucleosides, amino acids and other metabolites that are essential to cellular functions including innate immunity.

The stimulation of PRRs triggers cell signaling events resulting, among other consequences, in metabolic reprogramming supporting innate immune response. This has been best studied in immune cells although the molecular mechanisms have not been completely elucidated and greatly vary according to species and cell type. In macrophages and myeloid dendritic cells (DCs), Toll-like receptors (TLRs) stimulation modulates central carbon metabolism (**Figure 1**), resulting in increased glycolytic activity to support a pro-inflammatory phenotype (4–8). Glycolysis inhibition by 2-deoxy-glucose (2-DG) reduces the secretion of cytokines, the motility and the expression of costimulatory molecules that characterize mature DCs (5, 6, 8, 9). In murine macrophages and DCs, TLR4 stimulation by LPS is associated with a metabolic shift from OXPHOS to glycolysis despite the presence of oxygen (6, 10, 11). This shift is comparable to the Warburg-like effect in tumor cells. In human plasmacytoid DCs (pDCs), which are of lymphoid origin, TLR7 and TLR9 activation also stimulates glycolysis to support the production of type-I interferon (IFN-I) (11, 12). However, a recent study suggests that OXPHOS is also increased in these cells as opposed to myeloid DCs and necessary to support IFN-I production upon TLR7/9 engagement (13, 14). TLR4-induced type I IFN-β expression, was found to be dependent on the glycolysis and pentose phosphate pathway (15). Glycolysis activation mainly occurs *via* the upregulation of glycolytic enzymes, such as hexokinase 1 and 2 (HK1, HK2), glyceraldehyde-3-phosphate dehydrogenase (GAPDH), and pyruvate kinase isoenzyme M2 (PKM2). TLR engagement also increases the surface expression of the glucose transporter GLUT1 (16–18). This metabolic adaptation of macrophages, DCs and pDCs, is likely to support the synthesis of metabolites that are essential to immune functions, to satisfy energy needs for cell activation, and to allow immune cells to be functional even in oxygen-deprived environments. Moreover, glycolysis fuels the PPP to generate biosynthetic precursors for nucleotides, amino acids, and fatty acid synthesis (FAS), thereby supporting anabolic growth and cytokine secretion (19). Furthermore, the NADPH produced is used for the rapid production of microbicidal reactive oxygen species (ROS) by NADPH oxidase, and for glutathione regeneration, to maintain the redox balance.

Hexokinase (HK) activity is the rate-limiting enzyme controlling glucose entry into glycolysis (**Figure 1**). The glucose-6-phosphate produced can be either degraded by the glycolysis or serve as a precursor for ribose synthesis *via* the pentose phosphate pathway or glycogen production. TLR4 cell signaling induces both HK2 expression and phosphorylation in DCs by at least 2 pathways (20). In murine bone-marrow-derived DCs (BMDCs), TLR4 stimulation is known to induce the secretion of pro-inflammatory cytokines through p38-mitogen activated protein kinase (MAPK), c-Jun N-terminal kinase (JNK), nuclear factor kappa-B (NF-κB), but also activates TANK binding kinase 1 (TBK1)/inhibitor of NF-κB kinase subunit epsilon (IKKϵ) and protein kinase B/Akt that phosphorylates HK2 (5). This phosphorylation promotes HK2 binding to voltage-dependent anion channel (VDAC). Mitochondrial binding of HK2 is associated with enhanced glycolysis, reduced OXPHOS and resistance to apoptotic signals (21). The subcellular localization of HK2 dynamically regulates the catabolic versus anabolic fate of glucose-6-phosphate, promoting glycolysis when bound to the mitochondria and glycogen synthesis when located in the cytosol (22). LPS stimulation of murine BMDCs and macrophages through TLR4 also results, as a consequence of OXPHOS inhibition, in a broken TCA cycle. This leads to intracellular succinate accumulation that favors hypoxia induced factor (HIF)-1α stabilization (23, 24). HIF-1α induces the transcription of genes such as glycolytic enzymes, inducible nitric oxide synthase (iNOS) and pro-IL-1β. NO production by iNOS contributes to OXPHOS inhibition (4, 25). In human monocyte-derived DCs, p38-MAPK activation upon TLR4 engagement results in HIF-1α accumulation, enhancing the expression of glycolytic enzymes such as HK2 (8). Additionally, phosphatidylinositol-4,5-bisphosphate 3-kinase (PI3K)/Akt and mechanistic target of rapamycin (mTOR) signaling pathways are important modulators of cellular metabolism, sustaining anabolic metabolism and protein translation (26).

Intricate interactions between hepatitis viruses and TLRs have been reported with both activating and inhibiting effects (27, 28). At steady state, in primary human hepatocytes (PHH), only the expression of TLR3, 4 and 5 could be detected at the protein level. However, a much larger panel of TLRs was assessed in PHH by mRNA detection and activation by their cognate ligands, indicating that more TLRs are functional (29–31). In particular, it has been shown that HBV particles activate PHH through TLR2 (32), and HBV-infected PHH respond to TLR1/2 stimulation by the ligand Pam3CSK4 with an increasing sensitivity with time, suggesting a feedforward mechanism (33). HCV components can also be sensed by TLRs such as HCV Core that is recognized by human TLR2 (34). Conversely, hepatitis viruses have evolved countermeasures to block TLR signaling (28). For example, the NS3/4A protease of HCV is able to cleave TRIF, the signaling adaptor recruited by TLR3 (35). Another example is HBsAg from HBV that inhibits TLR3 and TLR4 activation in liver cells by their cognate ligands (36) and alters TLR2 response in macrophages (37). Finally, these complex interactions between hepatitis virus and TLR signaling can be manipulated for therapeutic purposes. For example, TLR1/2 and 3 ligands are investigated as a therapeutic approach to block HBV with direct antiviral effects on infected hepatocytes, whereas TLR7 and 8 ligands like GS-9620 and GS-9688 would act indirectly through the stimulation of other liver cell types such as pDC and macrophages (38). These interactions of hepatitis viruses with TLR signaling pathways probably have a direct impact on hepatocyte metabolism, but literature is surprisingly limited. Indeed, HBV interaction with TLR2 has been shown to increase LDL uptake and to induce the expression of low-density lipoprotein receptor (LDLR) and 3-hydroxy-3-methylglutharyl-coenzyme A reductase (HMGCR) in the hepatocyte cell line HepG2 *via* TLR2 (39). Interestingly, TLR2 also participates in metabolic reprogramming of CD8+ T cells and B cells in the woodchuck hepatitis virus (WHV) model and upon HBV stimulation (40, 41). Enhanced immune functions were associated to increased glucose consumption, lactate secretion and glutaminolysis, supporting a key role of this metabolic switch in the induction of an effective antiviral immune response. Altogether, this supports further studies to better explore the consequences of TLR engagement on liver metabolism in the context of hepatitis virus infections.

Detection of viral components by cytosolic PRRs also triggers metabolic reprogramming in liver cell types (29, 30). In particular, PHH are functional for cytosolic RNA sensors of the RIG-like receptor (RLR) family, retinoic acid-inducible gene I (RIG-I) and melanoma differentiation-associated gene 5 (MDA5), that trigger the downstream signaling molecule mitochondrial antiviral signaling protein (MAVS). RIG-I and MDA5 are essential in the sensing of HCV and HDV in hepatocytes (42–45). Several reports have recently established functional links between glucose metabolism and signaling pathways downstream of these receptors (46–48), which are usually associated with antiviral responses. On the one hand, glucose metabolism supports RIG-I and MDA5 signaling by feeding the hexosamine biosynthesis pathway that is required for the O-GlcNAcylation of MAVS (46). On the other hand, lactate produced by glycolysis inhibits RLR signaling by direct binding to MAVS (47). Besides, HK isoenzymes more directly interfere with RLR signaling in hepatocytes. Indeed, results obtained by Zhang W. et al. (47) indicated that HK2 interacts with both VDAC and MAVS. This mitochondrial localization stimulates HK2 activity and by increasing lactate production, inhibits MAVS signaling and restrains RIG-I-induced IFN-β secretion. Interestingly, RIG-I activation by viral RNAs dissociates HK2 from MAVS and thus reduces glycolysis and lactate production (47). Therefore, the reciprocal negative interactions between RLRs and HK2 form a toggle switch controlling innate immunity. Accordingly, we showed that HK2 expression but not HK4 (or GCK), the liver-specific hexokinase, inhibits RIG-I-induced IFN response in hepatocytic cell lines (49). In the infected liver, the situation is even more complex because hepatitis viruses have evolved mechanisms to block this pathway. The HBV X protein (HBx) binds MAVS and blocks IFN-β induction in response to RIG-I/MDA5 ligands (50–52). Upon HCV infection, the NS3-NS4A cleaves MAVS but also the E3 ubiquitin ligase Riplet that activates RIG-I (53–58). The consequence of these viral countermeasures on the metabolic reprogramming induced by the RLR/MAVS pathway in the context of HCV, HBV and HDV infections are largely unexplored.

Another important PRR that regulates metabolism is the stimulator of IFN genes protein (STING). This protein is a universal receptor for cyclic dinucleotides (cGAMP and cCGMP) which plays a pivotal role in cytosolic DNA sensing cascades and immune activation in response to DNA viruses, mitochondrial damages and genotoxic stress. Cyclic dinucleotides, which are produced by the cyclic GMP-AMP synthase (cGAS) in response to cytosolic DNA, bind to STING at the ER. This promotes the recruitment of TBK1, which phosphorylates the transcription factor IRF3, resulting in the production of IFN-I. Recently, several studies have linked STING activation to metabolic pathways (59). The STING pathway was identified as a key player in mediating obesity-induced chronic low-grade inflammation (60). Interestingly, chronic activation of TBK1 has been shown to inhibit mTORC1 activity, leading to dysregulated cellular metabolism (60). This is suggesting that activation of the STING pathway may inhibit mTORC1 signaling. Conversely, Meade N. et al. have shown that by targeting the mTORC1/mTORC2 regulatory circuit, the F17 protein of poxviruses suppresses STING signaling (61), indicating that the STING pathway is controlled by mTOR and could be regulated by nutrient availability. In the liver, STING is mainly expressed in non-parenchymal cells (including Kupffer cells, sinusoidal endothelial cells and stellate cells) but is virtually absent from hepatocytes (62). As a consequence, HBV DNA sensing is ineffective in hepatocytes because STING expression is too low (63). However, components of the cGAS/STING pathway can be upregulated in obese patients, which could favor the sensing of hepatitis viruses and aggravate inflammatory processes (64). Even though, the virus can also avoid DNA detection by active mechanisms (65). For example, it was shown that HBV polymerase inhibits the sensing of cytosolic DNA by interfering with the ubiquitin-dependent activation of STING (66). Besides, HCV has been shown to inhibit STING-mediated IFN induction through expression of the viral protein NS4B (67, 68).

## Viral hepatitis infection interferes with host cell metabolism

Although it was observed a long time ago that cellular metabolism increases upon viral infection, especially glucose consumption (69, 70), this phenomenon was little studied for decades. It is only since the 2010s and the advent of metabolomics and fluxomics technologies that the scientific community investigated the interference of viruses with central carbon metabolism at the cellular level. In recent years, many viruses have been shown to stimulate the biosynthetic pathways necessary for their replication (for review see (71)). However, underlying molecular mechanisms are not fully described. Furthermore, even if viral replication can be effectively impaired by specific metabolic inhibitors, whether this also involves indirect effects on antiviral immunity remains an open question. Therefore, the characterization of molecular mechanisms selected by viruses to control metabolism appears as a way to identify new pathways controlling innate immunity.

Hepatotropic viruses are highly adapted to hepatocytes, which have *per se* a very specific central carbon metabolism to regulate the energy homeostasis of the whole organism. Indeed, hepatocytes control glycemia by storing glucose in the form of glycogen upon insulin signaling (glycogenesis) or by producing glucose through breaking down intracellular glycogen (glycogenolysis) or gluconeogenesis from pyruvate. The liver is also at the center of the lipoproteins metabolism that distributes lipids throughout the organism. Hepatocytes have the ability to produce triglyceride-rich very-low density lipoproteins (VLDL) using lipids that are either neosynthesized from carbon sources or recycled from uptake of circulating lipoproteins. Hepatocytes are supplied by exogenous lipids coming from the intestine through chylomicron uptake during immediate post-prandial phases. Then, hepatocytes redistribute lipids in the form of VLDL that are secreted in the blood during inter-prandial phases. Thus, these cells have intrinsic specific capacities to switch their metabolism from anabolism to catabolism, depending on the body’s energy supply and demand. Therefore, chronic viral infection of hepatocytes requires specific viral strategies to control this unique cellular metabolism. For HCV, the interference with carbohydrate-lipid metabolism is exemplified by the metabolic syndrome developed in chronically infected patients (hypertension, insulin resistance, increased abdominal fat, dyslipidemia, steatosis and overweight). In chimpanzees, the most relevant *in vivo* model for HCV infection, a modulation of the genes involved in lipid metabolism was observed in animals that developed an acute infection and cleared or transiently cleared the infection (72). This was not the case in the animal that did not show an initial peak of viral replication but developed a persistent infection with a viral load only detected after 10 weeks. Upon HCV infection of hepatocytic cell lines, glucose consumption and STAT3 signaling pathway are increased and lipid peroxidation reduced (73), which correlates with accumulation of very long-chain fatty acids in cells and steatohepatitis in chronically-infected patients. Metabolomic combined with transcriptomic analyses in primary hepatocytes showed that metabolic pathways, including long chain fatty acid metabolism, glycolysis, and glycogen metabolism, are also altered by HBV (74). Although HBV is clearly inducing metabolic modulations in infected hepatocytes, HBV infection is probably not responsible for liver steatosis as opposed to HCV infection (75). It is thus clear that liver viruses interfere with host-specific cell metabolic pathways and we have reviewed below the major pathways that are targeted by HCV or HBV and its satellite virus HDV.

### Hepatitis virus interference with glucose metabolism

Like other viruses, HBV and HCV increase the glycolytic activity of infected cells (76–78) in different ways to support viral replication and virion production (77, 79, 80). Since intermediate metabolites of glycolysis are precursors for multiple biosynthetic pathways, it is not surprising that increasing glycolysis is paramount for viral particle synthesis. This increase in glycolytic activity is often associated with a decrease of oxidative phosphorylation, leading to the synthesis of lactate from pyruvate and thus promoting the flow of glycolytic intermediates required for anaplerosis. Likewise, during the cellular transformation of hepatocytes into cancer cells, one of the adaptations is the increase of glycolysis associated with reduced oxidative phosphorylation. It is now clear that by promoting the anabolic reactions necessary for its replication, HCV reprograms the metabolism of normal hepatocytes towards a profile similar to cancer cells (81, 82). By analyzing the proteome of HCV-infected cells, Diamond et al. first revealed in 2010 that HCV infection induces early perturbations in the glycolysis, that has repercussions on the pentose phosphate pathway and TCA cycle, which favor host biosynthetic activities supporting viral replication and propagation (83). In hepatoma cell lines, HCV decreases the expression of respiratory chain proteins, thus contributing to the fall of oxidative phosphorylation in the infected cell (84). The HCV-induced shift from oxidative phosphorylation to glycolysis appears to be dependent on activation of the nuclear factor HNF-4α (82). As previously reported in tumor cells, HCV induces pyruvate dehydrogenase kinase (PDK) activity which inhibits the entry of pyruvate into the TCA cycle, further promoting aerobic glycolysis (79). In this context, it was observed that HCV proteins expression activates HIF-1α leading to enhanced expression of glycolytic enzymes (76). The activation of HIF-1α was confirmed in liver biopsy specimens from patients with chronic hepatitis C. In Huh7.5 cells, the ectopic expression of HCV NS5A has the potential to induce insulin resistance by the phosphorylation of insulin receptor substrate (IRS)-1 at serine residue (Ser307) followed by decreased phosphorylation of Akt, forkhead box O1 (FoxO1) and glycogen synthase kinase 3 beta (GSK3β), the downstream players of insulin signaling pathway (85). In addition, the expression of the gluconeogenic enzyme phosphoenolpyruvate carboxykinase (PEPCK) and associated transcription factors are also up-regulated in hepatoma cells stably expressing NS5A or infected with HCV subgenomic replicon (86). In Huh7 cells, E2 expression was also a modulator of IRS-1 by impairing its insulin-induced phosphorylation and the phosphorylation of GSK3β, leading to an inhibition of glucose uptake and glycogen synthesis, respectively (87). It was also observed in HCV core transgenic mouse model that the core protein-induced serine phosphorylation of IRS-1 stimulates insulin resistance and decreases glucose uptake (88). Nevertheless, a high level of tumor necrosis factor-alpha, which has also been observed in human HCV patients, was considered to be one of the bases of insulin resistance in these transgenic mice. In hepatoma cell lines, the degradation of IRS-1 by HCV core protein translates to impaired ability of insulin to inhibit the expression of the target gene such as insulin growth factor binding protein-1 (IGFBP-1) and may provide a mechanism of insulin resistance and hyperglycemia observed in HCV patients (89). In HepG2 cell line, core protein expression results in the suppression of Sirtuin 1 expression at the origin of the upregulation of PEPCK and glucose-6-phosphate dehydrogenase (G6PD) and downregulation of glucose transporter 2 (GLUT2) (90, 91). The downregulation of cell surface expression of GLUT2 is also observed during infection (92). Meanwhile, we recently described that the HCV protein NS5A enhances glycolysis through a direct interaction with hexokinases, thereby altering the catalytic parameters of these enzymes (93, 94). Interestingly this enhancement of the glycolytic flux induced by NS5A does not result in the accumulation of glycogen suggesting a more complex rewiring of intracellular intermediate metabolites than expected in hepatocytes facing an increased glycolysis. Altogether, it suggests a complex interplay between viral replication and glycolytic control.

HBV infection or HBx expression in primary rat hepatocytes was also shown to alter glycolysis, and glycogen metabolism (74). In this cellular model, HBx was reported to activate mTORC1 and AMPK signaling, a master sensor of intracellular energy (95). These two factors have opposing effects on HBV replication and balance viral replication. Interestingly, HNF-4α has also been involved in the epigenetic regulation of glycolytic enzymes by HBV to meet the increased energy demand of infected cells (96). A truncated form of HBx called Ct-HBx was found to promote aerobic glycolysis by inhibiting the expression of thioredoxin-interacting protein (TXNIP) while increasing mTORC1 and HIF-1α expression (97). Proteomic analysis showed that cellular interactors of HBV whole-X protein (HBwx), a longer form of HBx, are functionally enriched in host proteins involved in glycolysis and gluconeogenesis (98). In hepatocytes, HBx protein also stimulates the expression of G6PD, the rate-limiting enzyme of the pentose phosphate pathway, in a Nrf2-dependent way (99). Altogether, these data suggest that HBV reprograms cellular glucose metabolism which may contribute to the development of HBV-associated hepatocarcinoma. Glycolysis and amino acid metabolism are also up-regulated in HCC cells transfected by the HBV core protein (HBc), suggesting that HBc contributes to the development of HCC (100). HBV surface glycoproteins were also involved in the metabolic reprogramming of hepatocytes. The large viral surface antigens of HBV (HBsAg-L) affects the oligomerization of pyruvate kinase isoform M2 (PKM2), thereby increasing glycolysis and lactate secretion (77). In addition, a natural mutant of HBsAg-L that is partially deleted for the pre-S2 region (pre-S2Delta) was reported to interact and activate the acid α-glucosidase, a lysosomal enzyme essential for the degradation of glycogen to glucose (101). In transgenic mice liver, the expression of this pre-S2Delta mutant can induce HCC and it initiates an mTOR-dependent glycolytic pathway contributing to glucose uptake and lactate production at the advanced stage of tumorigenesis (102). It should be stated that most of these results were obtained in various *in vitro* or *in vivo* models following overexpression of viral proteins, which imperfectly replicates physiological expression levels in the liver of infected patients (103).

### Hepatitis virus interference with lipid metabolism

A strong link between chronic HCV infection and lipid metabolism was discovered early because of liver steatosis associated to chronic HCV infection (104), the dysregulation of lipoprotein metabolism in patients (105) and because highly infectious particles are lipo-viral particles (LVP) that have the buoyant density of lipoproteins (106). Production of HCV viral particles requires the lipoprotein synthesis and secretion pathways (107) and cytoplasmic lipid droplets (LDs) are used by the virus as a platform of assembly (108, 109). Accordingly, LDs abundance, in which triglycerides are stored, is an essential determinant of HCV particle production (110–112). HCV infection up-regulates rate-limiting enzymes for cholesterol and fatty acids biosynthesis (113). Several proteins essential for LDs biogenesis, such as diacylglycerol acyltransferase (DGAT)-1 or perilipins are important host factors for HCV particles production (114–117). Because most infectious HCV viral particles are LVP that resemble lipoproteins, and because LDs are essential for lipidation of VLDL, it has been proposed that LDs are used in the first stages of infectious virions production (118). Lipoproteins are cargoes of neutral lipids (i.e. triglycerides and cholesterol esters) structured by proteins called apolipoproteins, which play an important role in the biology of circulating lipoproteins. While most apolipoproteins are exchangeable between lipoproteins in the blood stream, apolipoprotein B (ApoB) is structuring the particle and remains a lipoprotein constituent from its synthesis as a VLDL until its recapture by the liver. As LVP assembly depends on lipoprotein synthesis and secretion, important efforts have been made to elucidate how apolipoproteins interfere with HCV life cycle. Indeed, LVP share with VLDL several components such as ApoE, B, CI and CIII (119–124). In cell models, structural viral components such as capsid or envelope proteins associate with VLDL-precursor particles that form in the endoplasmic reticulum intermembrane space. Then luminal LDs bearing ApoE and ApoC can fuse with these precursor particles to form LVPs, in a similar process to one occurring during VLDL synthesis and maturation in hepatocytes. It was also demonstrated that viral envelopes can directly interact with ApoE reinforcing the intriguing interference of LVP morphogenesis with lipoproteins components (124). Moreover, ApoE is also involved in the interaction of LVP with scavenger receptor class B type 1 (SR-B1), LDLR, VLDL receptor and heparan sulfate proteoglycans (HSPGs), promoting HCV entry in hepatocytes (125–127). Interestingly it was recently reported that ApoE is enriched on the HBV envelope and promotes HBV infection and production (128). Targeting LDLR, both with blocking monoclonal antibody or siRNA, inhibits HBV infection (129). Targeting ApoE expression also interferes with the secretion of enveloped HBV particles, but not with non-enveloped nucleocapsid suggesting a role of ApoE in the secretion of infectious HBV particles. Despite this incorporation of ApoE into HBV viral envelope, HBV does not appear to induce intracellular lipid accumulation as does HCV. Detailed lipidomic analysis of HCV-infected hepatoma cells showed that HCV infection induced changes in the lipid composition of membranes and revealed that elevated polyunsaturated fatty acids were needed for virion morphogenesis (130). In liver cell lines replicating the virus, an accumulation of intracellular LDs was induced upon hypoxia, promoting the assembly of very low density, triglyceride-rich, highly infectious particles, similar to LVPs circulating in patients (131). These results establish a link between cellular respiration, response to hypoxia, and the synthesis of HCV particles through the modulation of lipid metabolism.

The expression of several HCV proteins was found to induce either LDs accumulation or *de novo* triglyceride synthesis (132, 133). Numerous studies found that HCV core protein associates to cellular lipid storage droplets both in liver biopsies from chronically HCV-infected chimpanzees and in various hepatocytic cell models (104, 134–136). HCV core protein expression alone is able to induce liver steatosis (105, 137–139) and the accumulation of larger or modified LDs in cultured hepatocytes (140–144). LDs association of HCV Core protein was found to determine the production of HCV infectious particles (145–147). HCV-NS5A can colocalize with Core at the surface of LDs (148, 149) and this is also important for viral particles production (150). HCV-NS5A protein upregulates fatty acid synthase (FAS) expression and therefore promotes synthesis of triglycerides (151). This induction depends on the AMPK/SREBP-1c signaling pathway (152). HCV RNA interacts with DEAD box polypeptide 3, X-linked (DDX3X) through its 3’ untranslated region, activating IKK-α, which translocates to the nucleus and induces a CBP/p300-mediated transcriptional program involving sterol regulatory element-binding proteins (SREBPs). This induces lipogenic genes and enhances core-associated LD formation to facilitate viral assembly (153).

HBV is also inducing major changes in lipid metabolism of hepatocytes (75). In the liver of HBV transgenic mice, the expression of genes involved in lipid synthesis is increased (154, 155). Chronic HBV infection associates with lipoproteins disorders included decreased circulating high density lipoproteins (HDL) and ApoA levels. HBV inhibits the transcription and translation of ApoA5, an apolipoprotein involved in the regulation of lipid metabolism, through its core gene (156). HBV also inhibits the synthesis and secretion of ApoC3 both *in vivo* and *in vitro* (157). It was found that HBx expression alone can cause lipid accumulation in hepatocytes, likely mediated by SREBP1 and peroxisome proliferator-activated receptor γ (PPARγ) (158). HBx can also increase the expression of liver fatty acid binding protein 1 (FABP1), a key driver gene of lipid accumulation in hepatoma cells, which level was enhanced in the sera of HBV-infected patients and the sera and liver of HBV transgenic mice (159). The induction of the steatogenic factors SREBP-1c, FAS, and PPAR can be triggered by liver-X-receptor (LXR) which is induced by HBx *in vitro* and in transgenic mice and is increased in human HBV-associated HCC (160). Intracellular lipids are also a source of energy that can be mobilized to fuel mitochondrial activity. Fatty acid oxidation (FAO) in the mitochondria produces acetyl-CoA that will enter the TCA cycle, fueling OXPHOS and generating large amounts of ATP. It was found that HBx interacts with essential enzymes for lipid metabolism (161) and activates the FAO upon glucose deprivation of cells and therefore plays a critical role for the survival of HBV-induced HCC cells (162). HBV also enhances nicotinamide phosphoribosyltransferase (NAMPT) expression, a critical rate-limiting enzyme involved in NAD synthesis, to support viral replication (163). NAD acts in the various metabolic signaling pathways as a coenzyme, and notably in fatty acid oxidation. NAD synthesis is also crucial to survival and proliferation of cancer cells.

Hepatocytes are also the cells that synthesize bile acids from cholesterol. Bile acid secretion into the intestine is primordial to fat emulsion before being absorbed by enterocytes. 90% of secreted bile acids are indeed reabsorbed and recycled through enterohepatic transport. Both HBV and HDV are indirectly dependent on bile salt metabolism for their infectivity, since they use as a receptor the bile acid transporter sodium taurocholate co-transporting polypeptide (NTCP), a transmembrane protein highly expressed in human hepatocytes (164). Later on, in the HDV replication cycle, an essential step in the virus assembly process involves the post-translational prenylation of the large isoform of the delta antigen (HDAg-L), introducing a lipid moiety (farnesyl) derived from the mevalonate-isoprenoid-cholesterol pathway. Preventing prenylation effectively abolishes virus particle formation (165). Therefore, farnesyl transferase inhibitors have been developed and Lonafarnib is now evaluated in advanced clinical trials to treat HDV-infected patients (166). Bile acids are also ligands of transcription factors such as farnesoid-X-receptor alpha (FXR). It was demonstrated that bile acids activate *in vitro* HCV replication through FXR (167, 168). Moreover, FXR heterodimers with retinoid X receptor alpha (RXR) can bind two sequences in the cccDNA of HBV, located in enhancer II and core promoter transcription regulatory regions (169). Indeed, activation of FXR by ligands resulted in inhibition of HBV infection in *in vitro* differentiated HepaRG cells and PHH, as well as in an *in vivo* mouse model (170). It was also found that a specific agonist of RXR inhibited HBV infection while knockdown of RXRα expression enhanced viral infection (171). This inhibitory effect on early-stage HBV infection is mediated by arachidonic acid and prostanoids. Inversely, it has been shown that HBV infection induces changes in FXR expression and activity with modifications of FXR target genes expression in a humanized mouse model and in liver biopsies from chronically infected patients (172). Finally, HBc protein expression enhanced the cholesterol biosynthesis pathway and inhibited the cholesterol degradation pathway in synergy with ethanol (173).

**Figure 2:**
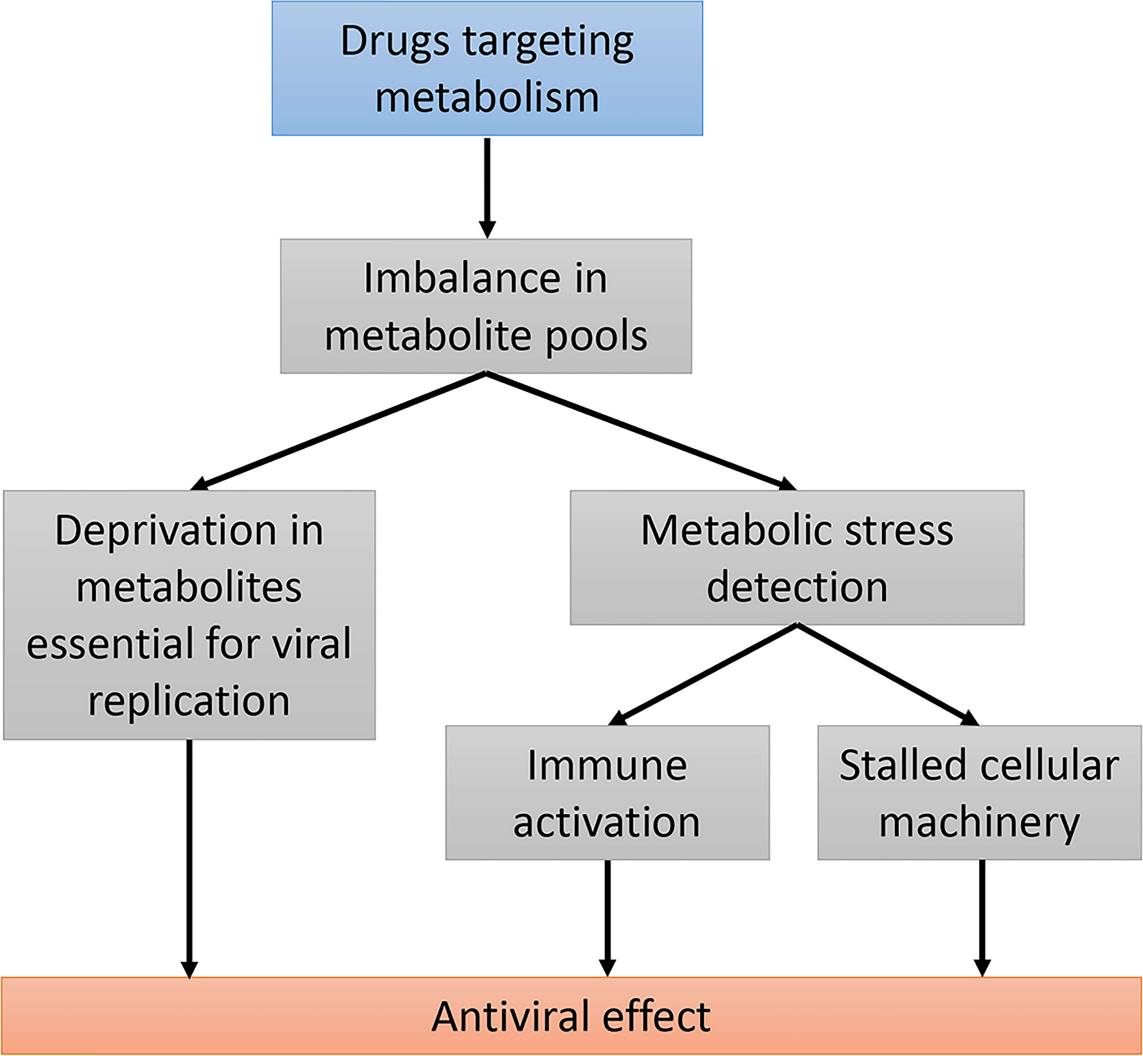
Using drugs targeting host metabolic pathways as an antiviral strategy. Modifying host cell metabolism using drugs will alter the balance in several metabolite pools. This can primarily prevent viral replication by depletion in essential metabolites. These metabolic changes can be detected by cellular metabolic sensors and have an impact on both the innate immunity response and the cellular machinery. Stalled cellular machinery could both enhance the antiviral immune response and prevent viral propagation.

All these results show that hepatitis viruses have a significant impact on hepatocyte metabolism. This can be due to the active reprogramming of specific pathways by viral proteins through molecular interactions. This may also simply reflect the adaptation of host cell metabolism to viral replication that consumes metabolites and energy in large quantities. A third mechanism is the activation of innate immunity pathways by viruses which is known to trigger metabolic changes as described above. When studying infected hepatocytes, we usually observe the overall result of these different interactions, and we only partially understand their specific contributions in the metabolic changes induced by hepatitis viruses. Finally, this necessarily has consequences on the innate immune response of infected hepatocytes. This is due to the complex interplay between cellular metabolism and innate immunity as described in part 1. This suggests that modulation of central metabolic pathways by HBV and HCV not only serves to meet the demand in basic metabolites during viral propagation, but also contributes to the inhibition of the innate antiviral response (**Figure 1**). It is also tempting to speculate that the glycolytic switch induced by these viruses promotes the expression of inflammatory cytokines contributing to disease progression. Moreover, enhanced aerobic glycolysis and lactate accumulation in this context may further inhibit MAVS activation, as described in a non-infectious context (47), thereby restraining the activation of the anti-viral RIG-I/MAVS pathway. Altogether, this supports the idea that metabolic pathway modulators could be used to starve hepatitis viruses from metabolites they need but also to restore the immune response in order to clear the infection (**Figure 2**). In the following chapter of this review, we provide evidence supporting this concept for the development of innovative therapies against hepatitis viruses.

## Stimulation of the innate antiviral response in hepatocytes with metabolic drugs

Since cellular metabolism is connected to the innate antiviral response in hepatocytes, this functional interaction gives leverage for developing innovative host-directed therapies. Drugs interfering with nucleoside/nucleotide biosynthesis well illustrate this idea since these antimetabolites enhance the innate antiviral response. A good example is provided by inhibitors of inosine monophosphate dehydrogenase (IMPDH), an enzyme converting inosine monophosphate into xanthine monophosphate in the purine nucleotide biosynthesis pathway. IMPDH inhibition with mycophenolic acid (MPA) has been shown to inhibit HCV replication in Huh7 cells both *in vitro* and in xenografted mice (174). IMPDH was also identified as a prominent target for inhibiting hepatitis E virus (HEV) replication (175). Indeed, MPA and other IMPDH inhibitors potently restrain the replication of HEV in Huh7 cells. Antiviral properties of MPA were associated to the induction of antiviral interferon-stimulated genes (ISGs) and synergistic effects with IFN-α stimulation (174, 175). Although initially controversial, recent reports showed that this induction of ISGs is reversed by guanosine supplementation of culture medium, thus demonstrating the role of purine depletion in MPA-treated cells (175). Besides, ISG induction is mediated by a non-canonical pathway that is independent of both IFN-I induction and JAK/STAT signaling. It has been suggested that a similar mechanism contributes to the anti-HCV effect of ribavirin, another inhibitor of IMPDH that is acting synergistically with IFN-α (176). However, these observations are apparently specific to transformed cells as a recent report showed that in PHH and in the non-transformed cell line HepaRG, ribavirin does not induce but rather represses and resets the expression of ISGs by chromatin remodeling (177). Besides, this phenomenon appears to be independent of IMPDH inhibition. Whether MPA is also showing the same activity in PHH and HepaRG cells has to be determined. More recently, it has been shown that an excess of guanosine also inhibits HCV replication (178). Indeed, when guanosine is added to the culture medium, intracellular levels of NDPs and NTPs are modified, thus increasing the frequency of mutations in HCV genome during viral replication. Quite unexpectedly, it was also found that the inhibition of enzymes upstream of IMPDH in the purine biosynthesis pathway does not inhibit but rather enhances the replication of HEV (175). Altogether, these results demonstrate that drugs inducing imbalance in the pool of purine nucleoside/nucleotide have an impact on the expression of ISGs, and this clearly contributes to their antiviral effect. However, the mechanisms involved need to be further investigated in primary cell cultures.

Conflicting results have been reported regarding the effect of purine biosynthesis inhibitors on HBV replication. MPA has been shown to enhance the replication of HBV in human hepatocyte cell lines HepG2 and Huh7 (179, 180). This effect is dependent on p38-MAPK and is reversed by the addition of guanosine in the culture medium, demonstrating the role of purine inhibition in this phenomenon (180). Conversely, an inhibitory effect or no effect were reported by other groups when treating HBV-replicating cell lines with MPA or VX-497, another IMPDH inhibitor (181–183). Most importantly, MPA was shown to inhibit HBV replication in PHH that represents the most relevant *in vitro* model for HBV infection (184). In these cells, the inhibitory effect of MPA was reversed by the addition of guanosine. Besides, MPA showed no antiviral effect in liver transplanted patients with HBV (185). Therefore, the inhibition of purine biosynthesis has variable effects on HBV infection depending on the cellular model and the metabolic status of host cells. Whether modulation of the innate immune response contributes to the impact of MPA on HBV replication has not been investigated yet.

The pyrimidine biosynthesis pathway was also involved in the replication of hepatitis viruses. Dihydroorotate dehydrogenase (DHODH) is the fourth rate-limiting enzyme in the *de novo* pyrimidine biosynthesis pathway and represents a prime target for pharmacological drugs. DHODH inhibition has been shown to block the replication of HCV in Huh7.5 or Huh7.5.1 cells (186, 187). Quite similarly, DHODH inhibition impaired the replication of HEV in Huh7 cells (175). Interestingly, DHODH inhibition with different pharmacological drugs has been shown to induce the expression of several ISGs through an IFN-independent and JAK/STAT-independent pathway (175). This induction was reversed by the addition of uridine, thus demonstrating that pyrimidine depletion is responsible for this induction of innate antiviral genes. The inhibition of orotidine-5’-monophosphate decarboxylase (ODCase), an enzyme that is two steps downstream of DHODH in the *de novo* pyrimidine biosynthesis pathway, showed a similar effect on ISG induction and HEV inhibition (175). In a similar fashion, the pyrimidine biosynthesis inhibitor Gemcitabine also impaired the replication of HEV through the activation of STAT1 and the induction of ISGs through a non-canonical pathway (188). Surprisingly, DHODH inhibitors Leflunomide and FK778 were initially reported to enhance the replication of HBV when tested in HepG2 and Huh7 cells, and this was confirmed in a recent report (179). However, the inhibition of the carbamoyl-phosphate synthetase 2, aspartate transcarbamylase and dihydroorotase (CAD) enzyme, which is upstream of DHODH in the pyrimidine biosynthesis pathway, showed no effect on HBV replication in HepAD38 or HepG2 cell lines (189). As above, this suggests that inhibitors of pyrimidine biosynthesis have variable effects on HBV that are highly context and cell type dependent. Finally, the same report showed that the CAD inhibitor PALA suppresses the replication of HDV both in Huh-106 and PHH (189). This antiviral effect of PALA is reversed by the addition of uridine to culture medium, thus demonstrating that it depends on pyrimidine biosynthesis inhibition. It is thus expected that DHODH inhibitors would also be able to inhibit HDV replication, but this has not yet been experimentally validated.

Besides nucleoside/nucleotide biosynthesis pathways, glucose metabolism also represents a therapeutic target to enhance the immune response against hepatitis viruses. Besides the already described O-GlcNAcylation of MAVS (46), hexosamine biosynthetic pathway positively regulates host antiviral response against HBV *in vitro* and *in vivo* through O-GlcNAc modification of sterile alpha motif and histidine/aspartic acid domain-containing protein 1 (SAMHD1) (190). Indeed, SAMHD1 is an interferon-induced deoxynucleotide triphosphate triphosphohydrolase (dNTPase) that restricts the replication of DNA viruses including HBV by degrading the intracellular pool of dNTPs. The O-GlcNAcylation of SAMHD1 stabilizes the expression of this restriction factor and increases its antiviral activity. These results reveal a link between the hexosamine pathway derived from the fructose-6-phosphate of the glycolytic pathway and innate antiviral immunity.

Other lipid-related factors that are targeted for antiviral purposes in the liver include components of the bile acid pathways, especially the bile acid transporter NTCP and the nuclear receptor FXR. Some links have been unraveled between bile acids metabolism and innate immunity. NTCP is a membrane transporter for bile acid but also an entry receptor for HBV and HDV that share the same surface glycoprotein HBs. Bulevirtide, a NTCP inhibitor derived from the preS1 peptide of HBs, is now used in the treatment of chronic HDV infection. Interestingly, it has been shown that by binding NTCP, Bulevirtide reverts the inhibitory effect of bile acids on the interferon response. This suggests that Bulevirtide inhibits HDV propagation not only by interfering with viral entry but also by restoring innate immunity (191). Since it has been proposed that FXR is hijacked by HBV to regulate viral transcription in cooperation with PGC-1α and SIRT1 (192), FXR modulators are now developed as HBV inhibitors and are evaluated in clinical trials. Whether the antiviral activity of FXR ligands on HBV infection implies a modulation of the immune response remains to be determined, but recent studies showed that FXR regulates inflammation. A direct interaction between FXR and NF-κB has been described resulting in a negative crosstalk between FXR and NF-κB signaling pathways. It has also been shown that treatment with FXR agonists inhibit the expression of inflammatory mediators in response to NF-κB activation in both HepG2 cells and *in vitro* cultured primary hepatocytes (193, 194). This nuclear receptor is also a negative regulator of NLRP3 inflammasome with trans-repressive effects on NF-κB and AP-1 target gene expression (195). Therefore, FXR ligands may have dual effects both on the virus and the innate immune response, and should help control the inflammatory response associated with chronic hepatitis. Like bile acids, vitamin D biosynthesis depends on cholesterol metabolism. Interestingly, vitamin D exhibits antiviral effects on HCV and this activity is associated with the induction and the amplification of IFN-I signaling (196, 197). Another cholesterol derivative and female sex hormone, 17β-estradiol, has been shown to protect cells from HCV infection and this antiviral effect depends on IFN-I induction (198). Altogether, these observations suggest that cholesterol metabolism in the liver could be targeted to inhibit hepatitis viruses through the modulation of innate immunity pathways.

Other metabolic pathways have been implicated in the regulation of antiviral immunity against several viruses. Although their impact on hepatitis viral infections has not been investigated yet, their broad antiviral activity deserves attention. This includes itaconate and its isomers metaconate and citraconate that were recently reported to inhibit the production of influenza virus particles by A549 infected cells (199). Immunomodulatory properties of itaconate have been extensively reviewed elsewhere (200). Interestingly, the TCA cycle intermediate succinate was also described to inhibit influenza virus infection both *in vitro* and *in vivo* by succinylation of the viral nucleoprotein (201). The potential impact of these metabolites on hepatitis virus infections deserves to be analyzed. Other metabolic pathways of prime interest include the tryptophan/kynurenine pathway. Although kynurenine is a well-known immunosuppressive metabolite, its degradation by kynurenine-3-monooxygenase (KMO) and downstream enzymes produces quinolinic acid that shows potent antiviral effects. Quinolinic acid has been shown to activate the N-methyl-D-aspartate receptor (NMDAR), which triggers Ca2+ influx, the phosphorylation of calcium/calmodulin-dependent protein kinase II (CaMKII) and IRF3, and finally leads to IFN-I production (202). This study shows that quinolinic acid produced from tryptophan has broad antiviral activity against HSV-1, adenovirus 5, VSV, influenza virus, ZIKV, DENV and SARS-CoV2. Whether it could also inhibit hepatitis viruses was not investigated.

## Conclusion

The development of drugs targeting host cellular metabolism as antivirals is an attractive strategy to deprive viruses of the metabolites they need and, more generally, to make the cellular environment inappropriate for viral replication (**Figure 2**). Targeting metabolism can also induce cellular stress that shuts down basic machinery such as protein translation, with indirect inhibitory effects on viral replication. As discussed in this review, a third possibility is that altered metabolism is detected by metabolic sensors that activate the innate immune response (**Figure 2**). This fits the interesting concept of “homeostasis-altering molecular processes” (HAMPs) developed by A. Liston and S.L. Masters in the context of inflammasome activation (203). Indeed, metabolic imbalance induced by specific viruses and drugs can be viewed as HAMPs, and quite similarly to PAMPs (pathogen-associated molecular patterns) and DAMPs (damage-associated molecular patterns), stimulate the immune response. Thus, targeting metabolic pathways to stimulate host innate immunity now appears to be a valuable strategy for controlling the replication of viruses. Modulators of nucleotide biosynthesis pathways have proven, for example, their ability to inhibit hepatitis viruses while stimulating the innate immune response. The exact contribution of immunity factors in the antiviral effect of these drugs needs to be further explored, and whether this general concept can be translated *in vivo* has to be determined. Lipid and cholesterol biosynthesis pathways are also considered as therapeutic targets in the treatment of HBV based on observations that drugs inhibiting these pathways decrease the production of subviral and/or viral particles (75, 204). Interestingly, serum lipid profiles change in HCV-infected patients treated with direct-acting antivirals (DAA) and this correlates with viral clearance (205, 206). Although direct evidence is missing, it is tempting to speculate that modulation of lipid metabolism associated to DAA contributes to viral clearance (207). In the case of HBV and HDV, metabolism could be manipulated on purpose with drugs to improve viral inhibition, to restore the immune response and finally to achieve functional cure. This will deserve attention in the near future and viral hepatitis, because of the close connection between viral replication, metabolism and innate immunity in the liver, is clearly an appropriate field to explore this concept.

## Author contributions

OD, P-OV, CR, VL, LP-C wrote sections of the manuscript. All authors contributed to the article and approved the submitted version.

## Funding

This work was supported by the Agence Nationale de Recherches sur le Sida et les Hépatites Virales Grants ASA21007CRA, ECTZ72972, ECTZ136480 and the Fondation pour la Recherche Médicale Grant DEQ20160334893.

## Conflict of interest

The authors declare that the research was conducted in the absence of any commercial or financial relationships that could be construed as a potential conflict of interest.

## Publisher’s note

All claims expressed in this article are solely those of the authors and do not necessarily represent those of their affiliated organizations, or those of the publisher, the editors and the reviewers. Any product that may be evaluated in this article, or claim that may be made by its manufacturer, is not guaranteed or endorsed by the publisher.

## References

[1] Muskiet FAJ Carrera-Bastos P Pruimboom L Lucia A Furman D. Obesity and leptin resistance in the regulation of the type I interferon early response and the increased risk for severe COVID-19. Nutrients (2022) 14:1388. doi: 10.3390/nu14071388 35406000 PMC9002648

[2] Zhou L He R Fang P Li M Yu H Wang Q. Hepatitis b virus rigs the cellular metabolome to avoid innate immune recognition. Nat Commun (2021) 12:98. doi: 10.1038/s41467-020-20316-8 33397935 PMC7782485

[3] Perrin-Cocon L Diaz O Aublin-Gex A Vidalain P-O Lotteau V. Reprogramming of central carbon metabolism in myeloid cells upon innate immune receptor stimulation. Immuno (2021) 1:1–14. doi: 10.3390/immuno1010001

[4] Everts B Amiel E van der Windt GJW Freitas TC Chott R Yarasheski KE. Commitment to glycolysis sustains survival of NO-producing inflammatory dendritic cells. Blood (2012) 120:1422–31. doi: 10.1182/blood-2012-03-419747 PMC3423780 22786879

[5] Everts B Amiel E Huang SC Smith AM Chang CH Lam WY. TLR-driven early glycolytic reprogramming *via* the kinases TBK1-IKKepsilon supports the anabolic demands of dendritic cell activation. Nat Immunol (2014) 15:323–32. doi: 10.1038/ni.2833 PMC4358322 24562310

[6] Krawczyk CM Holowka T Sun J Blagih J Amiel E DeBerardinis RJ. Toll-like receptor-induced changes in glycolytic metabolism regulate dendritic cell activation. Blood (2010) 115:4742–9. doi: 10.1182/blood-2009-10-249540 PMC2890190 20351312

[7] Perrin-Cocon L Aublin-Gex A Sestito SE Shirey KA Patel MC Andre P. TLR4 antagonist FP7 inhibits LPS-induced cytokine production and glycolytic reprogramming in dendritic cells, and protects mice from lethal influenza infection. Sci Rep (2017) 7:40791. doi: 10.1038/srep40791 28106157 PMC5247753

[8] Perrin-Cocon L Aublin-Gex A Diaz O Ramiere C Peri F Andre P. Toll-like receptor 4-induced glycolytic burst in human monocyte-derived dendritic cells results from p38-dependent stabilization of HIF-1alpha and increased hexokinase II expression. J Immunol (2018) 201:1510–21. doi: 10.4049/jimmunol.1701522 30037846

[9] Guak H Al Habyan S Ma EH Aldossary H Al-Masri M Won SY. Glycolytic metabolism is essential for CCR7 oligomerization and dendritic cell migration. Nat Commun (2018) 9:2463. doi: 10.1038/s41467-018-04804-6 29941886 PMC6018630

[10] Vijayan V Pradhan P Braud L Fuchs HR Gueler F Motterlini R. Human and murine macrophages exhibit differential metabolic responses to lipopolysaccharide - a divergent role for glycolysis. Redox Biol (2019) 22:101147. doi: 10.1016/j.redox.2019.101147 30825774 PMC6396203

[11] Bajwa G DeBerardinis RJ Shao B Hall B Farrar JD Gill MA. Cutting edge: Critical role of glycolysis in human plasmacytoid dendritic cell antiviral responses. J Immunol (2016) 196:2004–9. doi: 10.4049/jimmunol.1501557 PMC4761472 26826244

[12] Fekete T Sütö MI Bencze D Mázló A Szabo A Biro T. Human plasmacytoid and monocyte-derived dendritic cells display distinct metabolic profile upon RIG-I activation. Front Immunol (2018) 9:3070. doi: 10.3389/fimmu.2018.03070 30622542 PMC6308321

[13] Hurley HJ Dewald H Rothkopf ZS Singh S Jenkins F Deb P. Frontline science: AMPK regulates metabolic reprogramming necessary for interferon production in human plasmacytoid dendritic cells. J Leukoc Biol (2021) 109:299–308. doi: 10.1002/JLB.3HI0220-130 32640499

[14] Basit F Mathan T Sancho D de Vries IJM. Human dendritic cell subsets undergo distinct metabolic reprogramming for immune response. Front Immunol (2018) 9:2489. doi: 10.3389/fimmu.2018.02489 30455688 PMC6230993

[15] Erlich JR To EE Luong R Liong F Liong S Oseghale O. Glycolysis and the pentose phosphate pathway promote LPS-induced NOX2 oxidase- and IFN-β-Dependent inflammation in macrophages. Antioxidants (2022) 11:1488. doi: 10.3390/antiox11081488 36009206 PMC9405479

[16] Palsson-McDermott EM Curtis AM Goel G Lauterbach MA Sheedy FJ Gleeson LE. Pyruvate kinase M2 regulates hif-1alpha activity and IL-1beta induction and is a critical determinant of the warburg effect in LPS-activated macrophages. Cell Metab (2015) 21:65–80. doi: 10.1016/j.cmet.2014.12.005 25565206 PMC5198835

[17] Moon J-S Hisata S Park M-A DeNicola GM Ryter SW Nakahira K. mTORC1-induced HK1-dependent glycolysis regulates NLRP3 inflammasome activation. Cell Rep (2015) 12:102–15. doi: 10.1016/j.celrep.2015.05.046 PMC4858438 26119735

[18] Chang C-H Curtis JD Maggi LB Faubert B Villarino AV O’Sullivan D. Posttranscriptional control of T cell effector function by aerobic glycolysis. Cell (2013) 153:1239–51. doi: 10.1016/j.cell.2013.05.016 PMC3804311 23746840

[19] O’Neill LA Kishton RJ Rathmell J. A guide to immunometabolism for immunologists. Nat Rev Immunol (2016) 16:553–65. doi: 10.1038/nri.2016.70 PMC5001910 27396447

[20] Perrin-Cocon L Aublin-Gex A Lotteau V. Metabolic reprogramming of myeloid cells upon TLR4 stimulation. In: Rossetti C Peri F, editors. The role of toll-like receptor 4 in infectious and non infectious inflammation. Cham: Springer International Publishing (2021). p. 159–74. doi: 10.1007/978-3-030-56319-6_11

[21] Roberts DJ Tan-Sah VP Smith JM Miyamoto S. Akt phosphorylates HK-II at thr-473 and increases mitochondrial HK-II association to protect cardiomyocytes. J Biol Chem (2013) 288:23798–806. doi: 10.1074/jbc.M113.482026 PMC3745326 23836898

[22] John S Weiss JN Ribalet B. Subcellular localization of hexokinases I and II directs the metabolic fate of glucose. PloS One (2011) 6:e17674. doi: 10.1371/journal.pone.0017674 21408025 PMC3052386

[23] Tannahill GM Curtis AM Adamik J Palsson-McDermott EM McGettrick AF Goel G. Succinate is an inflammatory signal that induces IL-1beta through HIF-1alpha. Nature (2013) 496:238–42. doi: 10.1038/nature11986 PMC4031686 23535595

[24] Mills E O’Neill LA. Succinate: a metabolic signal in inflammation. Trends Cell Biol (2014) 24:313–20. doi: 10.1016/j.tcb.2013.11.008 24361092

[25] Palmieri EM Gonzalez-Cotto M Baseler WA Davies LC Ghesquière B Maio N. Nitric oxide orchestrates metabolic rewiring in M1 macrophages by targeting aconitase 2 and pyruvate dehydrogenase. Nat Commun (2020) 11:698. doi: 10.1038/s41467-020-14433-7 32019928 PMC7000728

[26] Saravia J Raynor JL Chapman NM Lim SA Chi H. Signaling networks in immunometabolism. Cell Res (2020) 30:328–42. doi: 10.1038/s41422-020-0301-1 PMC7118125 32203134

[27] Redwan EM Aljadawi AA Uversky VN. Hepatitis c virus infection and intrinsic disorder in the signaling pathways induced by toll-like receptors. Biology (2022) 11:1091. doi: 10.3390/biology11071091 36101469 PMC9312352

[28] You H Qin S Zhang F Hu W Li X Liu D. Regulation of pattern-recognition receptor signaling by HBX during hepatitis b virus infection. Front Immunol (2022) 13:829923. doi: 10.3389/fimmu.2022.829923 35251017 PMC8891514

[29] Faure-Dupuy S Vegna S Aillot L Dimier L Esser K Broxtermann M. Characterization of pattern recognition receptor expression and functionality in liver primary cells and derived cell lines. J Innate Immun (2018) 10:339–48. doi: 10.1159/000489966 PMC6757176 29975940

[30] Nicolay W Moeller R Kahl S Vondran FWR Pietschmann T Kunz S. Characterization of RNA sensing pathways in hepatoma cell lines and primary human hepatocytes. Cells (2021) 10:3019. doi: 10.3390/cells10113019 34831243 PMC8616302

[31] Broering R Lutterbeck M Trippler M Kleinehr K Poggenpohl L Paul A. Long-term stimulation of toll-like receptor 3 in primary human hepatocytes leads to sensitization for antiviral responses induced by poly I:C treatment. J Viral Hepat (2014) 21:480–90. doi: 10.1111/jvh.12174 24750363

[32] Zhang Z Trippler M Real CI Werner M Luo X Schefczyk S. Hepatitis b virus particles activate toll-like receptor 2 signaling initially upon infection of primary human hepatocytes. Hepatology (2020) 72:829–44. doi: 10.1002/hep.31112 31925967

[33] Desmares M Delphin M Chardès B Pons C Riedinger J Michelet M. Insights on the antiviral mechanisms of action of the TLR1/2 agonist Pam3CSK4 in hepatitis b virus (HBV)-infected hepatocytes. Antiviral Res (2022) 206:105386. doi: 10.1016/j.antiviral.2022.105386 35963549

[34] Chang S Dolganiuc A Szabo G. Toll-like receptors 1 and 6 are involved in TLR2-mediated macrophage activation by hepatitis c virus core and NS3 proteins. J leukocyte Biol (2007) 82:479–87. doi: 10.1189/jlb.0207128 17595379

[35] Li K Foy E Ferreon JC Nakamura M Ferreon ACM Ikeda M. Immune evasion by hepatitis c virus NS3/4A protease-mediated cleavage of the toll-like receptor 3 adaptor protein TRIF. Proc Natl Acad Sci USA (2005) 102:2992–7. doi: 10.1073/pnas.0408824102 PMC548795 15710891

[36] Wu J Meng Z Jiang M Pei R Trippler M Broering R. Hepatitis b virus suppresses toll-like receptor-mediated innate immune responses in murine parenchymal and nonparenchymal liver cells. Hepatology (2009) 49:1132–40. doi: 10.1002/hep.22751 19140219

[37] Wang S Chen Z Hu C Qian F Cheng Y Wu M. Hepatitis b virus surface antigen selectively inhibits TLR2 ligand-induced IL-12 production in monocytes/macrophages by interfering with JNK activation. J Immunol (2013) 190:5142–51. doi: 10.4049/jimmunol.1201625 23585678

[38] Lucifora J Bonnin M Aillot L Fusil F Maadadi S Dimier L. Direct antiviral properties of TLR ligands against HBV replication in immune-competent hepatocytes. Sci Rep (2018) 8:5390. doi: 10.1038/s41598-018-23525-w 29599452 PMC5876392

[39] Li Y-J Zhu P Liang Y Yin W-G Xiao J-H. Hepatitis b virus induces expression of cholesterol metabolism-related genes *via* TLR2 in HepG2 cells. World J Gastroenterol (2013) 19:2262–9. doi: 10.3748/wjg.v19.i14.2262 PMC3627892 23599654

[40] Li Q Wang J Islam H Kirschning C Lu H Hoffmann D. Hepatitis b virus particles activate b cells through the TLR2-MyD88-mTOR axis. Cell Death Dis (2021) 12:34. doi: 10.1038/s41419-020-03284-1 33414473 PMC7791069

[41] Zhang E Ma Z Li Q Yan H Liu J Wu W. TLR2 stimulation increases cellular metabolism in CD8+ T cells and thereby enhances CD8+ T cell activation, function, and antiviral activity. J Immunol (2019) 203:2872–86. doi: 10.4049/jimmunol.1900065 31636238

[42] Sumpter R Loo Y-M Foy E Li K Yoneyama M Fujita T. Regulating intracellular antiviral defense and permissiveness to hepatitis c virus RNA replication through a cellular RNA helicase, RIG-I. J Virol (2005) 79:2689–99. doi: 10.1128/JVI.79.5.2689-2699.2005 PMC548482 15708988

[43] Saito T Hirai R Loo Y-M Owen D Johnson CL Sinha SC. Regulation of innate antiviral defenses through a shared repressor domain in RIG-I and LGP2. Proc Natl Acad Sci U.S.A. (2007) 104:582–7. doi: 10.1073/pnas.0606699104 PMC1766428 17190814

[44] Israelow B Narbus CM Sourisseau M Evans MJ. HepG2 cells mount an effective antiviral interferon-lambda based innate immune response to hepatitis c virus infection. Hepatology (2014) 60:1170–9. doi: 10.1002/hep.27227 PMC4176518 24833036

[45] Zhang Z Filzmayer C Ni Y Sültmann H Mutz P Hiet M-S. Hepatitis d virus replication is sensed by MDA5 and induces IFN-β/λ responses in hepatocytes. J Hepatol (2018) 69:25–35. doi: 10.1016/j.jhep.2018.02.021 29524530

[46] Li T Li X Attri KS Liu C Li L Herring LE. O-GlcNAc transferase links glucose metabolism to MAVS-mediated antiviral innate immunity. Cell Host Microbe (2018) 24:791–803.e6. doi: 10.1016/j.chom.2018.11.001 30543776 PMC6296827

[47] Zhang W Wang G Xu ZG Tu H Hu F Dai J. Lactate is a natural suppressor of RLR signaling by targeting MAVS. Cell (2019) 178:176–189 e15. doi: 10.1016/j.cell.2019.05.003 31155231 PMC6625351

[48] Cao Z Zhou Y Zhu S Feng J Chen X Liu S. Pyruvate carboxylase activates the RIG-i-like receptor-mediated antiviral immune response by targeting the MAVS signalosome. Sci Rep (2016) 6:22002. doi: 10.1038/srep22002 26906558 PMC4764940

[49] Perrin-Cocon L Vidalain P-O Jacquemin C Aublin-Gex A Olmstead K Panthu B. A hexokinase isoenzyme switch in human liver cancer cells promotes lipogenesis and enhances innate immunity. Commun Biol (2021) 4:217. doi: 10.1038/s42003-021-01749-3 33594203 PMC7886870

[50] Kumar M Jung SY Hodgson AJ Madden CR Qin J Slagle BL. Hepatitis b virus regulatory HBx protein binds to adaptor protein IPS-1 and inhibits the activation of beta interferon. J Virol (2011) 85:987–95. doi: 10.1128/JVI.01825-10 PMC3020017 21068253

[51] Wang X Li Y Mao A Li C Li Y Tien P. Hepatitis b virus X protein suppresses virus-triggered IRF3 activation and IFN-beta induction by disrupting the VISA-associated complex. Cell Mol Immunol (2010) 7:341–8. doi: 10.1038/cmi.2010.36 PMC4002683 20711230

[52] Wei C Ni C Song T Liu Y Yang X Zheng Z. The hepatitis b virus X protein disrupts innate immunity by downregulating mitochondrial antiviral signaling protein. J Immunol (2010) 185:1158–68. doi: 10.4049/jimmunol.0903874 20554965

[53] Li X-D Sun L Seth RB Pineda G Chen ZJ. Hepatitis c virus protease NS3/4A cleaves mitochondrial antiviral signaling protein off the mitochondria to evade innate immunity. Proc Natl Acad Sci (2005) 102:17717–22. doi: 10.1073/pnas.0508531102 PMC1308909 16301520

[54] Loo Y-M Owen DM Li K Erickson AK Johnson CL Fish PM. Viral and therapeutic control of IFN-β promoter stimulator 1 during hepatitis c virus infection. Proc Natl Acad Sci (2006) 103:6001–6. doi: 10.1073/pnas.0601523103 PMC1458687 16585524

[55] Bellecave P Sarasin-Filipowicz M Donzé O Kennel A Gouttenoire J Meylan E. Cleavage of mitochondrial antiviral signaling protein in the liver of patients with chronic hepatitis c correlates with a reduced activation of the endogenous interferon system. Hepatology (2010) 51:1127–36. doi: 10.1002/hep.23426 20044805

[56] Meylan E Curran J Hofmann K Moradpour D Binder M Bartenschlager R. Cardif is an adaptor protein in the RIG-I antiviral pathway and is targeted by hepatitis c virus. Nature (2005) 437:1167–72. doi: 10.1038/nature04193 16177806

[57] Oshiumi H Miyashita M Matsumoto M Seya T. A distinct role of riplet-mediated K63-linked polyubiquitination of the RIG-I repressor domain in human antiviral innate immune responses. PloS Pathog (2013) 9:e1003533. doi: 10.1371/journal.ppat.1003533 23950712 PMC3738492

[58] Foy E Li K Wang C Sumpter R Ikeda M Lemon SM. Regulation of interferon regulatory factor-3 by the hepatitis c virus serine protease. Science (2003) 300:1145–8. doi: 10.1126/science.1082604 12702807

[59] Bai J Liu F. The cGAS-cGAMP-STING pathway: A molecular link between immunity and metabolism. Diabetes (2019) 68:1099–108. doi: 10.2337/dbi18-0052 PMC6610018 31109939

[60] Hasan M Gonugunta VK Dobbs N Ali A Palchik G Calvaruso MA. Chronic innate immune activation of TBK1 suppresses mTORC1 activity and dysregulates cellular metabolism. Proc Natl Acad Sci U.S.A. (2017) 114:746–51. doi: 10.1073/pnas.1611113114 PMC5278463 28069950

[61] Meade N Furey C Li H Verma R Chai Q Rollins MG. Poxviruses evade cytosolic sensing through disruption of an mTORC1-mTORC2 regulatory circuit. Cell (2018) 174:1143–1157.e17. doi: 10.1016/j.cell.2018.06.053 30078703 PMC6172959

[62] Thomsen MK Nandakumar R Stadler D Malo A Valls RM Wang F. Lack of immunological DNA sensing in hepatocytes facilitates hepatitis b virus infection. Hepatology (2016) 64:746–59. doi: 10.1002/hep.28685 27312012

[63] Lauterbach-Rivière L Bergez M Mönch S Qu B Riess M Vondran FWR. Hepatitis b virus DNA is a substrate for the cGAS/STING pathway but is not sensed in infected hepatocytes. Viruses (2020) 12:E592. doi: 10.3390/v12060592 PMC7354540 32485908

[64] Donne R Saroul-Ainama M Cordier P Hammoutene A Kabore C Stadler M. Replication stress triggered by nucleotide pool imbalance drives DNA damage and cGAS-STING pathway activation in NAFLD. Dev Cell (2022) 57:1728–1741.e6. doi: 10.1016/j.devcel.2022.06.003 35768000

[65] Chen H Jiang L Chen S Hu Q Huang Y Wu Y. HBx inhibits DNA sensing signaling pathway *via* ubiquitination and autophagy of cGAS. Virol J (2022) 19:55. doi: 10.1186/s12985-022-01785-3 35346247 PMC8962493

[66] Liu Y Li J Chen J Li Y Wang W Du X. Hepatitis b virus polymerase disrupts K63-linked ubiquitination of STING to block innate cytosolic DNA-sensing pathways. J Virol (2015) 89:2287–300. doi: 10.1128/JVI.02760-14 PMC4338878 25505063

[67] Nitta S Sakamoto N Nakagawa M Kakinuma S Mishima K Kusano-Kitazume A. Hepatitis c virus NS4B protein targets STING and abrogates RIG-i-mediated type I interferon-dependent innate immunity. Hepatology (2013) 57:46–58. doi: 10.1002/hep.26017 22911572

[68] Ding Q Cao X Lu J Huang B Liu Y-J Kato N. Hepatitis c virus NS4B blocks the interaction of STING and TBK1 to evade host innate immunity. J Hepatol (2013) 59:52–8. doi: 10.1016/j.jhep.2013.03.019 23542348

[69] Levy HB Baron S. The effect of animal viruses on host cell metabolism II. effect of poliomyelitis virus on GLycolysis and uptake of glycine by monkey kidney tissue cultures. J Infect Dis (1957) 100:109–18. doi: 10.1093/infdis/100.2.109 13416643

[70] Henle G Deinhardt F Bergs VV Henle W. Studies on persistent infections of tissue cultures: I. general aspects of the system. J Exp Med (1958) 108:537–60. doi: 10.1084/jem.108.4.537 PMC2136904 13575683

[71] Mayer KA Stöckl J Zlabinger GJ Gualdoni GA. Hijacking the supplies: Metabolism as a novel facet of virus-host interaction. Front Immunol (2019) 10:1533. doi: 10.3389/fimmu.2019.01533 31333664 PMC6617997

[72] Su AI Pezacki JP Wodicka L Brideau AD Supekova L Thimme R. Genomic analysis of the host response to hepatitis c virus infection. Proc Natl Acad Sci U.S.A. (2002) 99:15669–74. doi: 10.1073/pnas.202608199 PMC137774 12441396

[73] Lupberger J Croonenborghs T Roca Suarez AA Van Renne N Jühling F Oudot MA. Combined analysis of metabolomes, proteomes, and transcriptomes of hepatitis c virus–infected cells and liver to identify pathways associated with disease development. Gastroenterology (2019) 157:537–551.e9. doi: 10.1053/j.gastro.2019.04.003 30978357 PMC8318381

[74] Lamontagne RJ Casciano JC Bouchard MJ. A broad investigation of the HBV-mediated changes to primary hepatocyte physiology reveals HBV significantly alters metabolic pathways. Metabolism (2018) 83:50–9. doi: 10.1016/j.metabol.2018.01.007 PMC5960616 29410347

[75] Zhang J Ling N Lei Y Peng M Hu P Chen M. Multifaceted interaction between hepatitis b virus infection and lipid metabolism in hepatocytes: A potential target of antiviral therapy for chronic hepatitis b. Front Microbiol (2021) 12:636897. doi: 10.3389/fmicb.2021.636897 33776969 PMC7991784

[76] Ripoli M D’Aprile A Quarato G Sarasin-Filipowicz M Gouttenoire J Scrima R. Hepatitis c virus-linked mitochondrial dysfunction promotes hypoxia-inducible factor 1 alpha-mediated glycolytic adaptation. J Virol (2010) 84:647–60. doi: 10.1128/JVI.00769-09 PMC2798449 19846525

[77] Wu Y-H Yang Y Chen C-H Hsiao C-J Li T-N Liao K-J. Aerobic glycolysis supports hepatitis b virus protein synthesis through interaction between viral surface antigen and pyruvate kinase isoform M2. PloS Pathog (2021) 17:e1008866. doi: 10.1371/journal.ppat.1008866 33720996 PMC8009439

[78] Wan Q Wang Y Tang H. Quantitative ^13^ c traces of glucose fate in hepatitis b virus-infected hepatocytes. Anal Chem (2017) 89:3293–9. doi: 10.1021/acs.analchem.6b03200 28221022

[79] Jung G-S Jeon J-H Choi Y-K Jang SY Park SY Kim S-W. Pyruvate dehydrogenase kinase regulates hepatitis c virus replication. Sci Rep (2016) 6:30846. doi: 10.1038/srep30846 27471054 PMC4965757

[80] Yu T Yang Q Tian F Chang H Hu Z Yu B. Glycometabolism regulates hepatitis c virus release. PloS Pathog (2021) 17:e1009746. doi: 10.1371/journal.ppat.1009746 34297778 PMC8301660

[81] Gerresheim GK Roeb E Michel AM Niepmann M. Hepatitis c virus downregulates core subunits of oxidative phosphorylation, reminiscent of the warburg effect in cancer cells. Cells (2019) 8:1410. doi: 10.3390/cells8111410 31717433 PMC6912740

[82] Levy G Habib N Guzzardi MA Kitsberg D Bomze D Ezra E. Nuclear receptors control pro-viral and antiviral metabolic responses to hepatitis c virus infection. Nat Chem Biol (2016) 12:1037–45. doi: 10.1038/nchembio.2193 PMC7046487 27723751

[83] Diamond DL Syder AJ Jacobs JM Sorensen CM Walters K-A Proll SC. Temporal proteome and lipidome profiles reveal hepatitis c virus-associated reprogramming of hepatocellular metabolism and bioenergetics. PloS Pathog (2010) 6:e1000719. doi: 10.1371/journal.ppat.1000719 20062526 PMC2796172

[84] Gerresheim GK Bathke J Michel AM Andreev DE Shalamova LA Rossbach O. Cellular gene expression during hepatitis c virus replication as revealed by ribosome profiling. Int J Mol Sci (2019) 20:E1321. doi: 10.3390/ijms20061321 PMC6470931 30875926

[85] Parvaiz F Manzoor S Iqbal J Sarkar-Dutta M Imran M Waris G. Hepatitis c virus NS5A promotes insulin resistance through IRS-1 serine phosphorylation and increased gluconeogenesis. World J Gastroenterol (2015) 21:12361–9. doi: 10.3748/wjg.v21.i43.12361 PMC4649119 26604643

[86] Qadri I Choudhury M Rahman SM Knotts TA Janssen RC Schaack J. Increased phosphoenolpyruvate carboxykinase gene expression and steatosis during hepatitis c virus subgenome replication: role of nonstructural component 5A and CCAAT/enhancer-binding protein β. J Biol Chem (2012) 287:37340–51. doi: 10.1074/jbc.M112.384743 PMC3481331 22955269

[87] Hsieh M-J Lan K-P Liu H-Y Zhang X-Z Lin Y-F Chen T-Y. Hepatitis c virus E2 protein involve in insulin resistance through an impairment of Akt/PKB and GSK3β signaling in hepatocytes. BMC Gastroenterol (2012) 12:74. doi: 10.1186/1471-230X-12-74 22721429 PMC3464126

[88] Shintani Y Fujie H Miyoshi H Tsutsumi T Tsukamoto K Kimura S. Hepatitis c virus infection and diabetes: direct involvement of the virus in the development of insulin resistance. Gastroenterology (2004) 126:840–8. doi: 10.1053/j.gastro.2003.11.056 14988838

[89] Alberstein M Zornitzki T Zick Y Knobler H. Hepatitis c core protein impairs insulin downstream signalling and regulatory role of IGFBP-1 expression. J Viral Hepat (2012) 19:65–71. doi: 10.1111/j.1365-2893.2011.01447.x 22187946

[90] Li Z Gu X Hu J Ping Y Li H Yan J. Hepatitis c virus core protein impairs metabolic disorder of liver cell *via* HOTAIR-Sirt1 signalling. Bioscience Rep (2016) 36:e00336. doi: 10.1042/BSR20160088 PMC5293566 27129296

[91] Yu J-W Sun L-J Liu W Zhao Y-H Kang P Yan B-Z. Hepatitis c virus core protein induces hepatic metabolism disorders through down-regulation of the SIRT1-AMPK signaling pathway. Int J Infect Dis (2013) 17 :e539–45. doi: 10.1016/j.ijid.2013.01.027 23510538

[92] Kasai D Adachi T Deng L Nagano-Fujii M Sada K Ikeda M. HCV replication suppresses cellular glucose uptake through down-regulation of cell surface expression of glucose transporters. J Hepatol (2009) 50:883–94. doi: 10.1016/j.jhep.2008.12.029 19303158

[93] Ramiere C Rodriguez J Enache LS Lotteau V Andre P Diaz O. Activity of hexokinase is increased by its interaction with hepatitis c virus protein NS5A. J Virol (2014) 88:3246–54. doi: 10.1128/JVI.02862-13 PMC3957934 24390321

[94] Perrin-Cocon L Kundlacz C Jacquemin C Hanoulle X Aublin-Gex A Figl M. Domain 2 of hepatitis c virus protein NS5A activates glucokinase and induces lipogenesis in hepatocytes. Int J Mol Sci (2022) 23:919. doi: 10.3390/ijms23020919 35055105 PMC8780509

[95] Bagga S Rawat S Ajenjo M Bouchard MJ. Hepatitis b virus (HBV) X protein-mediated regulation of hepatocyte metabolic pathways affects viral replication. Virology (2016) 498:9–22. doi: 10.1016/j.virol.2016.08.006 27529294

[96] Sengupta I Mondal P Sengupta A Mondal A Singh V Adhikari S. Epigenetic regulation of fructose-1,6-bisphosphatase 1 by host transcription factor speckled 110 kDa during hepatitis b virus infection. FEBS J (2022) 289 :6694–713. doi: 10.1111/febs.16544 35653238

[97] Zhang Y Yan Q Gong L Xu H Liu B Fang X. C-terminal truncated HBx initiates hepatocarcinogenesis by downregulating TXNIP and reprogramming glucose metabolism. Oncogene (2021) 40:1147–61. doi: 10.1038/s41388-020-01593-5 PMC7878188 33323975

[98] Zhang Y Liu J Liu H He Y Yi R Niu Y. Comparative study of the different activities of hepatitis b virus whole-X protein and HBx in hepatocarcinogenesis by proteomics and bioinformatics analysis. Arch Virol (2015) 160:1645–56. doi: 10.1007/s00705-015-2421-3 25913689

[99] Liu B Fang M He Z Cui D Jia S Lin X. Hepatitis b virus stimulates G6PD expression through HBx-mediated Nrf2 activation. Cell Death Dis (2015) 6:e1980. doi: 10.1038/cddis.2015.322 26583321 PMC4670929

[100] Xie Q Fan F Wei W Liu Y Xu Z Zhai L. Multi-omics analyses reveal metabolic alterations regulated by hepatitis b virus core protein in hepatocellular carcinoma cells. Sci Rep (2017) 7:41089. doi: 10.1038/srep41089 28112229 PMC5253728

[101] Hung J-H Yan C-W Su I-J Wang H-C Lei H-Y Lin W-C. Hepatitis b virus surface antigen interacts with acid alpha-glucosidase and alters glycogen metabolism. Hepatol Res (2010) 40:633–40. doi: 10.1111/j.1872-034X.2010.00645.x 20618459

[102] Teng C-F Hsieh W-C Wu H-C Lin Y-J Tsai H-W Huang W. Hepatitis b virus pre-S2 mutant induces aerobic glycolysis through mammalian target of rapamycin signal cascade. PloS One (2015) 10:e0122373. doi: 10.1371/journal.pone.0122373 25909713 PMC4409318

[103] Slagle BL Andrisani OM Bouchard MJ Lee CGL Ou J-HJ Siddiqui A. Technical standards for hepatitis b virus X protein (HBx) research. Hepatology (2015) 61:1416–24. doi: 10.1002/hep.27360 PMC4320676 25099228

[104] Roingeard P Hourioux C. Hepatitis c virus core protein, lipid droplets and steatosis. J Viral Hepat (2008) 15:157–64. doi: 10.1111/j.1365-2893.2007.00953.x 18086178

[105] Perlemuter G Sabile A Letteron P Vona G Topilco A Chretien Y. Hepatitis c virus core protein inhibits microsomal triglyceride transfer protein activity and very low density lipoprotein secretion: a model of viral-related steatosis. FASEB J (2002) 16:185–94. doi: 10.1096/fj.01-0396com 11818366

[106] Andre P Komurian-Pradel F Deforges S Perret M Berland JL Sodoyer M. Characterization of low- and very-low-density hepatitis c virus RNA-containing particles. J Virol (2002) 76:6919–28. doi: 10.1128/JVI.76.14.6919-6928.2002 PMC136313 12072493

[107] Icard V Diaz O Scholtes C Perrin-Cocon L Ramiere C Bartenschlager R. Secretion of hepatitis c virus envelope glycoproteins depends on assembly of apolipoprotein b positive lipoproteins. PloS One (2009) 4:e4233. doi: 10.1371/journal.pone.0004233 19156195 PMC2617766

[108] Roingeard P Hourioux C Blanchard E Prensier G. Hepatitis c virus budding at lipid droplet-associated ER membrane visualized by 3D electron microscopy. Histochem Cell Biol (2008) 130:561–6. doi: 10.1007/s00418-008-0447-2 18512067

[109] Ferraris P Beaumont E Uzbekov R Brand D Gaillard J Blanchard E. Sequential biogenesis of host cell membrane rearrangements induced by hepatitis c virus infection. Cell Mol Life Sci (2013) 70:1297–306. doi: 10.1007/s00018-012-1213-0 PMC4901162 23184194

[110] Liefhebber JMP Hague CV Zhang Q Wakelam MJO McLauchlan J. Modulation of triglyceride and cholesterol ester synthesis impairs assembly of infectious hepatitis c virus*. J Biol Chem (2014) 289:21276–88. doi: 10.1074/jbc.M114.582999 PMC4118089 24917668

[111] Zhang J Lan Y Sanyal S. Modulation of lipid droplet metabolism-a potential target for therapeutic intervention in flaviviridae infections. Front Microbiol (2017) 8:2286. doi: 10.3389/fmicb.2017.02286 29234310 PMC5712332

[112] Galli A Ramirez S Bukh J. Lipid droplets accumulation during hepatitis c virus infection in cell-culture varies among genotype 1–3 strains and does not correlate with virus replication. Viruses (2021) 13:389. doi: 10.3390/v13030389 33671086 PMC7999684

[113] Sugiyama K Ebinuma H Nakamoto N Sakasegawa N Murakami Y Chu P-S. Prominent steatosis with hypermetabolism of the cell line permissive for years of infection with hepatitis c virus. PloS One (2014) 9:e94460. doi: 10.1371/journal.pone.0094460 24718268 PMC3981821

[114] Herker E Harris C Hernandez C Carpentier A Kaehlcke K Rosenberg AR. Efficient hepatitis c virus particle formation requires diacylglycerol acyltransferase-1. Nat Med (2010) 16:1295–8. doi: 10.1038/nm.2238 PMC3431199 20935628

[115] Vogt DA Camus G Herker E Webster BR Tsou C-L Greene WC. Lipid droplet-binding protein TIP47 regulates hepatitis c virus RNA replication through interaction with the viral NS5A protein. PloS Pathog (2013) 9:e1003302. doi: 10.1371/journal.ppat.1003302 23593007 PMC3623766

[116] Rösch K Kwiatkowski M Hofmann S Schöbel A Grüttner C Wurlitzer M. Quantitative lipid droplet proteome analysis identifies annexin A3 as a cofactor for HCV particle production. Cell Rep (2016) 16:3219–31. doi: 10.1016/j.celrep.2016.08.052 27653686

[117] Lassen S Gruttner C Nguyen-Dinh V Herker E. Perilipin-2 is critical for efficient lipoprotein and hepatitis c virus particle production. J Cell Sci (2019) 132 :jcs217042. doi: 10.1242/jcs.217042 30559250

[118] Bartenschlager R Penin F Lohmann V Andre P. Assembly of infectious hepatitis c virus particles. Trends Microbiol (2011) 19:95–103. doi: 10.1016/j.tim.2010.11.005 21146993

[119] Nielsen SU Bassendine MF Burt AD Martin C Pumeechockchai W Toms GL. Association between hepatitis c virus and very-low-density lipoprotein (VLDL)/LDL analyzed in iodixanol density gradients. J Virol (2006) 80:2418–28. doi: 10.1128/JVI.80.5.2418-2428.2006 PMC1395398 16474148

[120] Merz A Long G Hiet M-S Brügger B Chlanda P Andre P. Biochemical and morphological properties of hepatitis c virus particles and determination of their lipidome. J Biol Chem (2011) 286:3018–32. doi: 10.1074/jbc.M110.175018 PMC3024796 21056986

[121] Thomssen R Bonk S Propfe C Heermann KH Köchel HG Uy A. Association of hepatitis c virus in human sera with beta-lipoprotein. Med Microbiol Immunol (1992) 181:293–300. doi: 10.1007/BF00198849 1335546

[122] Scholtes C Ramière C Rainteau D Perrin-Cocon L Wolf C Humbert L. High plasma level of nucleocapsid-free envelope glycoprotein-positive lipoproteins in hepatitis c patients. Hepatology (2012) 56:39–48. doi: 10.1002/hep.25628 22290760

[123] Meunier J-C Russell RS Engle RE Faulk KN Purcell RH Emerson SU. Apolipoprotein c1 association with hepatitis c virus. J Virol (2008) 82:9647–56. doi: 10.1128/JVI.00914-08 PMC2546963 18667498

[124] Boyer A Dumans A Beaumont E Etienne L Roingeard P Meunier J-C. The association of hepatitis c virus glycoproteins with apolipoproteins e and b early in assembly is conserved in lipoviral particles. J Biol Chem (2014) 289:18904–13. doi: 10.1074/jbc.M113.538256 PMC4081931 24838241

[125] Agnello V Abel G Elfahal M Knight GB Zhang QX. Hepatitis c virus and other flaviviridae viruses enter cells *via* low density lipoprotein receptor. Proc Natl Acad Sci U.S.A. (1999) 96:12766–71. doi: 10.1073/pnas.96.22.12766 PMC23090 10535997

[126] Jiang J Wu X Tang H Luo G. Apolipoprotein e mediates attachment of clinical hepatitis c virus to hepatocytes by binding to cell surface heparan sulfate proteoglycan receptors. PloS One (2013) 8:e67982. doi: 10.1371/journal.pone.0067982 23844141 PMC3699494

[127] Libeu CP Lund-Katz S Phillips MC Wehrli S Hernáiz MJ Capila I. New insights into the heparan sulfate proteoglycan-binding activity of apolipoprotein e. J Biol Chem (2001) 276:39138–44. doi: 10.1074/jbc.M104746200 11500500

[128] Qiao L Luo GG. Human apolipoprotein e promotes hepatitis b virus infection and production. PloS Pathog (2019) 15:e1007874. doi: 10.1371/journal.ppat.1007874 31393946 PMC6687101

[129] Li Y Luo G. Human low-density lipoprotein receptor plays an important role in hepatitis b virus infection. PloS Pathog (2021) 17 :e1009722. doi: 10.1371/journal.ppat.1009722 34293069 PMC8345860

[130] Hofmann S Krajewski M Scherer C Scholz V Mordhorst V Truschow P. Complex lipid metabolic remodeling is required for efficient hepatitis c virus replication. Biochim Biophys Acta (BBA) - Mol Cell Biol Lipids (2018) 1863:1041–56. doi: 10.1016/j.bbalip.2018.06.002 29885363

[131] Cochard J Bull-Maurer A Tauber C Burlaud-Gaillard J Mazurier F Meunier J-C. Differentiated cells in prolonged hypoxia produce highly infectious native-like hepatitis c virus particles. Hepatology (2021) 74:627–40. doi: 10.1002/hep.31788 33665810

[132] Lerat H Kammoun HL Hainault I Merour E Higgs MR Callens C. Hepatitis c virus proteins induce lipogenesis and defective triglyceride secretion in transgenic mice. J Biol Chem (2009) 284:33466–74. doi: 10.1074/jbc.M109.019810 PMC2785191 19808675

[133] Nasheri N Joyce M Rouleau Y Yang P Yao S Tyrrell DL. Modulation of fatty acid synthase enzyme activity and expression during hepatitis c virus replication. Chem Biol (2013) 20:570–82. doi: 10.1016/j.chembiol.2013.03.014 23601646

[134] Barba G Harper F Harada T Kohara M Goulinet S Matsuura Y. Hepatitis c virus core protein shows a cytoplasmic localization and associates to cellular lipid storage droplets. Proc Natl Acad Sci U.S.A. (1997) 94:1200–5. doi: 10.1073/pnas.94.4.1200 PMC19768 9037030

[135] McLauchlan J Lemberg MK Hope G Martoglio B. Intramembrane proteolysis promotes trafficking of hepatitis c virus core protein to lipid droplets. EMBO J (2002) 21:3980–8. doi: 10.1093/emboj/cdf414 PMC126158 12145199

[136] Boulant S Montserret R Hope RG Ratinier M Targett-Adams P Lavergne J-P. Structural determinants that target the hepatitis c virus core protein to lipid droplets. J Biol Chem (2006) 281:22236–47. doi: 10.1074/jbc.M601031200 16704979

[137] Moriya K Yotsuyanagi H Shintani Y Fujie H Ishibashi K Matsuura Y. Hepatitis c virus core protein induces hepatic steatosis in transgenic mice. J Gen Virol (1997) 78(Pt 7):1527–31. doi: 10.1099/0022-1317-78-7-1527 9225025

[138] Moriya K Fujie H Shintani Y Yotsuyanagi H Tsutsumi T Ishibashi K. The core protein of hepatitis c virus induces hepatocellular carcinoma in transgenic mice. Nat Med (1998) 4:1065–7. doi: 10.1038/2053 9734402

[139] Harris C Herker E Farese RV Ott M. Hepatitis c virus core protein decreases lipid droplet turnover: A MECHANISM FOR CORE-INDUCED STEATOSIS*. J Biol Chem (2011) 286:42615–25. doi: 10.1074/jbc.M111.285148 PMC3234948 21984835

[140] Piodi A Chouteau P Lerat H Hézode C Pawlotsky J-M. Morphological changes in intracellular lipid droplets induced by different hepatitis c virus genotype core sequences and relationship with steatosis. Hepatology (2008) 48:16–27. doi: 10.1002/hep.22288 18570290

[141] Boulant S Douglas MW Moody L Budkowska A Targett-Adams P McLauchlan J. Hepatitis c virus core protein induces lipid droplet redistribution in a microtubule- and dynein-dependent manner. Traffic (2008) 9:1268–82. doi: 10.1111/j.1600-0854.2008.00767.x 18489704

[142] Lyn RK Kennedy DC Stolow A Ridsdale A Pezacki JP. Dynamics of lipid droplets induced by the hepatitis c virus core protein. Biochem Biophys Res Commun (2010) 399:518–24. doi: 10.1016/j.bbrc.2010.07.101 20678475

[143] Mazumder N Lyn RK Singaravelu R Ridsdale A Moffatt DJ Hu C-W. Fluorescence lifetime imaging of alterations to cellular metabolism by domain 2 of the hepatitis c virus core protein. PloS One (2013) 8:e66738. doi: 10.1371/journal.pone.0066738 23826122 PMC3691201

[144] Loizides-Mangold U Clément S Alfonso-Garcia A Branche E Conzelmann S Parisot C. HCV 3a core protein increases lipid droplet cholesteryl ester content *via* a mechanism dependent on sphingolipid biosynthesis. PloS One (2014) 9:e115309. doi: 10.1371/journal.pone.0115309 25522003 PMC4270764

[145] Shavinskaya A Boulant S Penin F McLauchlan J Bartenschlager R. The lipid droplet binding domain of hepatitis c virus core protein is a major determinant for efficient virus assembly. J Biol Chem (2007) 282:37158–69. doi: 10.1074/jbc.M707329200 17942391

[146] Boulant S Targett-Adams P McLauchlan J. Disrupting the association of hepatitis c virus core protein with lipid droplets correlates with a loss in production of infectious virus. J Gen Virol (2007) 88:2204–13. doi: 10.1099/vir.0.82898-0 17622624

[147] Dansako H Hiramoto H Ikeda M Wakita T Kato N. Rab18 is required for viral assembly of hepatitis c virus through trafficking of the core protein to lipid droplets. Virology (2014) 462–463:166–74. doi: 10.1016/j.virol.2014.05.017 24997429

[148] Camus G Herker E Modi AA Haas JT Ramage HR Farese RV. Diacylglycerol acyltransferase-1 localizes hepatitis c virus NS5A protein to lipid droplets and enhances NS5A interaction with the viral capsid core. J Biol Chem (2013) 288:9915–23. doi: 10.1074/jbc.M112.434910 PMC3617291 23420847

[149] Salloum S Wang H Ferguson C Parton RG Tai AW. Rab18 binds to hepatitis c virus NS5A and promotes interaction between sites of viral replication and lipid droplets. PloS Pathog (2013) 9:e1003513. doi: 10.1371/journal.ppat.1003513 23935497 PMC3731246

[150] Masaki T Suzuki R Murakami K Aizaki H Ishii K Murayama A. Interaction of hepatitis c virus nonstructural protein 5A with core protein is critical for the production of infectious virus particles. J Virol (2008) 82:7964–76. doi: 10.1128/JVI.00826-08 PMC2519576 18524832

[151] Yang W Hood BL Chadwick SL Liu S Watkins SC Luo G. Fatty acid synthase is up-regulated during hepatitis c virus infection and regulates hepatitis c virus entry and production. Hepatology (2008) 48:1396–403. doi: 10.1002/hep.22508 PMC2614928 18830996

[152] Meng Z Liu Q Sun F Qiao L. Hepatitis c virus nonstructural protein 5A perturbs lipid metabolism by modulating AMPK/SREBP-1c signaling. Lipids Health Dis (2019) 18:191. doi: 10.1186/s12944-019-1136-y 31684957 PMC6829953

[153] Li Q Pène V Krishnamurthy S Cha H Liang TJ. Hepatitis c virus infection activates an innate pathway involving IKK-α in lipogenesis and viral assembly. Nat Med (2013) 19:722–9. doi: 10.1038/nm.3190 PMC3676727 23708292

[154] Hajjou M Norel R Carver R Marion P Cullen J Rogler LE. cDNA microarray analysis of HBV transgenic mouse liver identifies genes in lipid biosynthetic and growth control pathways affected by HBV. J Med Virol (2005) 77:57–65. doi: 10.1002/jmv.20427 16032730

[155] Yang F Yan S He Y Wang F Song S Guo Y. Expression of hepatitis b virus proteins in transgenic mice alters lipid metabolism and induces oxidative stress in the liver. J Hepatol (2008) 48:12–9. doi: 10.1016/j.jhep.2007.06.021 18037187

[156] Zhu C Gao G Song H Xu F Wu K Liu X. Hepatitis b virus inhibits apolipoprotein A5 expression through its core gene. Lipids Health Dis (2016) 15:178. doi: 10.1186/s12944-016-0340-2 27724895 PMC5057420

[157] Zhu C Zhu H Song H Xu L Li L Liu F. Hepatitis b virus inhibits the *in vivo* and *in vitro* synthesis and secretion of apolipoprotein C3. Lipids Health Dis (2017) 16:213. doi: 10.1186/s12944-017-0607-2 29132372 PMC5683573

[158] Kim KH Shin H-J Kim K Choi HM Rhee SH Moon H-B. Hepatitis b virus X protein induces hepatic steatosis *Via* transcriptional activation of SREBP1 and PPARγ. Gastroenterology (2007) 132:1955–67. doi: 10.1053/j.gastro.2007.03.039 17484888

[159] Wu Y-L Peng X-E Zhu Y-B Yan X-L Chen W-N Lin X. Hepatitis b virus X protein induces hepatic steatosis by enhancing the expression of liver fatty acid binding protein. J Virol (2016) 90:1729–40. doi: 10.1128/JVI.02604-15 PMC4733992 26637457

[160] Na T-Y Shin YK Roh KJ Kang S-A Hong I Oh SJ. Liver X receptor mediates hepatitis b virus X protein–induced lipogenesis in hepatitis b virus–associated hepatocellular carcinoma. Hepatology (2009) 49:1122–31. doi: 10.1002/hep.22740 19105208

[161] Xu Z Zhai L Yi T Gao H Fan F Li Y. Hepatitis b virus X induces inflammation and cancer in mice liver through dysregulation of cytoskeletal remodeling and lipid metabolism. Oncotarget (2016) 7:70559–74. doi: 10.18632/oncotarget.12372 PMC5342574 27708241

[162] Wang M-D Wu H Huang S Zhang H-L Qin C-J Zhao L-H. HBx regulates fatty acid oxidation to promote hepatocellular carcinoma survival during metabolic stress. Oncotarget (2016) 7:6711–26. doi: 10.18632/oncotarget.6817 PMC4872744 26744319

[163] Guo H-J Li H-Y Chen Z-H Zhou W-J Li J-J Zhang J-Y. NAMPT promotes hepatitis b virus replication and liver cancer cell proliferation through the regulation of aerobic glycolysis. Oncol Lett (2021) 21:390. doi: 10.3892/ol.2021.12651 33777213 PMC7988713

[164] Yan H Zhong G Xu G He W Jing Z Gao Z. Sodium taurocholate cotransporting polypeptide is a functional receptor for human hepatitis b and d virus. Elife (2012) 1:e00049. doi: 10.7554/eLife.00049 23150796 PMC3485615

[165] Bordier BB Ohkanda J Liu P Lee S-Y Salazar FH Marion PL. *In vivo* antiviral efficacy of prenylation inhibitors against hepatitis delta virus. J Clin Invest (2003) 112:407–14. doi: 10.1172/JCI17704 PMC166292 12897208

[166] Yardeni D Heller T Koh C. Chronic hepatitis d-what is changing? J Viral Hepat (2022) 29:240–51. doi: 10.1111/jvh.13651 35122369

[167] Scholtes C Diaz O Icard V Kaul A Bartenschlager R Lotteau V. Enhancement of genotype 1 hepatitis c virus replication by bile acids through FXR. J Hepatol (2008) 48:192–9. doi: 10.1016/j.jhep.2007.09.015 18096266

[168] Chhatwal P Bankwitz D Gentzsch J Frentzen A Schult P Lohmann V. Bile acids specifically increase hepatitis c virus RNA-replication. PloS One (2012) 7:e36029. doi: 10.1371/journal.pone.0036029 22558311 PMC3338857

[169] Ramiere C Scholtes C Diaz O Icard V Perrin-Cocon L Trabaud MA. Transactivation of the hepatitis b virus core promoter by the nuclear receptor FXRalpha. J Virol (2008) 82:10832–40. doi: 10.1128/JVI.00883-08 PMC2573182 18768987

[170] Mouzannar K Fusil F Lacombe B Ollivier A Menard C Lotteau V. Farnesoid X receptor-alpha is a proviral host factor for hepatitis b virus that is inhibited by ligands *in vitro* and *in vivo* . FASEB J (2019) 33:2472–83. doi: 10.1096/fj.201801181R 30307769

[171] Song M Sun Y Tian J He W Xu G Jing Z. Silencing retinoid X receptor alpha expression enhances early-stage hepatitis b virus infection in cell cultures. J Virol (2018) 92:e01771-17. doi: 10.1128/JVI.01771-17 29437960 PMC5874418

[172] Oehler N Volz T Bhadra OD Kah J Allweiss L Giersch K. Binding of hepatitis b virus to its cellular receptor alters the expression profile of genes of bile acid metabolism. Hepatology (2014) 60:1483–93. doi: 10.1002/hep.27159 24711282

[173] Wang Y Wu T Hu D Weng X Wang X Chen P-J. Intracellular hepatitis b virus increases hepatic cholesterol deposition in alcoholic fatty liver *via* hepatitis b core protein. J Lipid Res (2018) 59:58–68. doi: 10.1194/jlr.M079533 29133292 PMC5748497

[174] Pan Q de Ruiter PE Metselaar HJ Kwekkeboom J de Jonge J Tilanus HW. Mycophenolic acid augments interferon-stimulated gene expression and inhibits hepatitis c virus infection *in vitro* and *in vivo* . Hepatology (2012) 55:1673–83. doi: 10.1002/hep.25562 22213147

[175] Wang Y Wang W Xu L Zhou X Shokrollahi E Felczak K. Cross talk between nucleotide synthesis pathways with cellular immunity in constraining hepatitis e virus replication. Antimicrob Agents Chemother (2016) 60:2834–48. doi: 10.1128/AAC.02700-15 PMC4862450 26926637

[176] Thomas E Feld JJ Li Q Hu Z Fried MW Liang TJ. Ribavirin potentiates interferon action by augmenting interferon-stimulated gene induction in hepatitis c virus cell culture models. Hepatology (2011) 53:32–41. doi: 10.1002/hep.23985 21254160 PMC3498496

[177] Testoni B Durantel D Lebossé F Fresquet J Helle F Negro F. Ribavirin restores IFNα responsiveness in HCV-infected livers by epigenetic remodelling at interferon stimulated genes. Gut (2016) 65:672–82. doi: 10.1136/gutjnl-2014-309011 26082258

[178] Sabariegos R Ortega-Prieto AM Díaz-Martínez L Grande-Pérez A Crespo CG Gallego I. Guanosine inhibits hepatitis c virus replication and increases indel frequencies, associated with altered intracellular nucleotide pools. PloS Pathog (2022) 18:e1010210. doi: 10.1371/journal.ppat.1010210 35085375 PMC8794218

[179] Ruan J Sun S Cheng X Han P Zhang Y Sun D. Mitomycin, 5-fluorouracil, leflunomide, and mycophenolic acid directly promote hepatitis b virus replication and expression *in vitro* . Virol J (2020) 17:89. doi: 10.1186/s12985-020-01339-5 32611423 PMC7331192

[180] Hoppe-Seyler K Sauer P Lohrey C Hoppe-Seyler F. The inhibitors of nucleotide biosynthesis leflunomide, FK778, and mycophenolic acid activate hepatitis b virus replication *in vitro* . Hepatology (2012) 56:9–16. doi: 10.1002/hep.25602 22271223

[181] Wu J Xie H-Y Jiang G-P Xu X Zheng S-S. The effect of mycophenolate acid on hepatitis b virus replication *in vitro* . Hepatobiliary Pancreat Dis Int (2003) 2:410–3. Available at: http://www.hbpdint.com/EN/Y2003/V2/I3/410 14599949

[182] Ying C De Clercq E Neyts J. Ribavirin and mycophenolic acid potentiate the activity of guanine- and diaminopurine-based nucleoside analogues against hepatitis b virus. Antiviral Res (2000) 48:117–24. doi: 10.1016/s0166-3542(00)00121-2 11114413

[183] Markland W McQuaid TJ Jain J Kwong AD. Broad-spectrum antiviral activity of the IMP dehydrogenase inhibitor VX-497: a comparison with ribavirin and demonstration of antiviral additivity with alpha interferon. Antimicrob Agents Chemother (2000) 44:859–66. doi: 10.1128/AAC.44.4.859-866.2000 PMC89783 10722482

[184] Gong ZJ De Meyer S Clarysse C Verslype C Neyts J De Clercq E. Mycophenolic acid, an immunosuppressive agent, inhibits HBV replication *in vitro* . J Viral Hepat (1999) 6:229–36. doi: 10.1046/j.1365-2893.1999.00163.x 10607235

[185] Ben-Ari Z Zemel R Tur-Kaspa R. The addition of mycophenolate mofetil for suppressing hepatitis b virus replication in liver recipients who developed lamivudine resistance–no beneficial effect. Transplantation (2001) 71:154–6. doi: 10.1097/00007890-200101150-00026 11211184

[186] Hoffmann H-H Kunz A Simon VA Palese P Shaw ML. Broad-spectrum antiviral that interferes with *de novo* pyrimidine biosynthesis. Proc Natl Acad Sci U.S.A. (2011) 108:5777–82. doi: 10.1073/pnas.1101143108 PMC3078400 21436031

[187] Yang Y Cao L Gao H Wu Y Wang Y Fang F. Discovery, optimization, and target identification of novel potent broad-spectrum antiviral inhibitors. J Med Chem (2019) 62:4056–73. doi: 10.1021/acs.jmedchem.9b00091 30938999

[188] Li Y Li P Li Y Zhang R Yu P Ma Z. Drug screening identified gemcitabine inhibiting hepatitis e virus by inducing interferon-like response *via* activation of STAT1 phosphorylation. Antiviral Res (2020) 184:104967. doi: 10.1016/j.antiviral.2020.104967 33137361

[189] Verrier ER Weiss A Bach C Heydmann L Turon-Lagot V Kopp A. Combined small molecule and loss-of-function screen uncovers estrogen receptor alpha and CAD as host factors for HDV infection and antiviral targets. Gut (2020) 69:158–67. doi: 10.1136/gutjnl-2018-317065 PMC6943243 30833451

[190] Hu J Gao Q Yang Y Xia J Zhang W Chen Y. Hexosamine biosynthetic pathway promotes the antiviral activity of SAMHD1 by enhancing O-GlcNAc transferase-mediated protein O-GlcNAcylation. Theranostics (2021) 11:805–23. doi: 10.7150/thno.50230 PMC7738853 33391506

[191] Verrier ER Colpitts CC Bach C Heydmann L Zona L Xiao F. Solute carrier NTCP regulates innate antiviral immune responses targeting hepatitis c virus infection of hepatocytes. Cell Rep (2016) 17:1357–68. doi: 10.1016/j.celrep.2016.09.084 PMC5098118 27783949

[192] Curtil C Enache LS Radreau P Dron AG Scholtes C Deloire A. The metabolic sensors FXRalpha, PGC-1alpha, and SIRT1 cooperatively regulate hepatitis b virus transcription. FASEB J (2014) 28:1454–63. doi: 10.1096/fj.13-236372 24297698

[193] Balasubramaniyan N Ananthanarayanan M Suchy FJ. Nuclear factor-κB regulates the expression of multiple genes encoding liver transport proteins. Am J Physiol Gastrointest Liver Physiol (2016) 310:G618–628. doi: 10.1152/ajpgi.00363.2015 PMC4836129 26867564

[194] Wang Y-D Chen W-D Wang M Yu D Forman BM Huang W. Farnesoid X receptor antagonizes nuclear factor kappaB in hepatic inflammatory response. Hepatology (2008) 48:1632–43. doi: 10.1002/hep.22519 PMC3056574 18972444

[195] Fiorucci S Biagioli M Zampella A Distrutti E. Bile acids activated receptors regulate innate immunity. Front Immunol (2018) 9:2018.01853. doi: 10.3389/fimmu.2018.01853 30150987 PMC6099188

[196] Gal-Tanamy M Bachmetov L Ravid A Koren R Erman A Tur-Kaspa R. Vitamin d: an innate antiviral agent suppressing hepatitis c virus in human hepatocytes. Hepatology (2011) 54:1570–9. doi: 10.1002/hep.24575 21793032

[197] Gutierrez JA Jones KA Flores R Singhania A Woelk CH Schooley RT. Vitamin d metabolites inhibit hepatitis c virus and modulate cellular gene expression. J Virol Antivir Res (2014) 3 :10.4172/2324-8955.1000129. doi: 10.4172/2324-8955.1000129 PMC4651454 26594646

[198] Barbaglia MN Harris JM Smirnov A Burlone ME Rigamonti C Pirisi M. 17β-oestradiol protects from hepatitis c virus infection through induction of type I interferon. Viruses (2022) 14:1806. doi: 10.3390/v14081806 36016428 PMC9415988

[199] Chen F Elgaher W a. M Winterhoff M Büssow K Waqas FH Graner E. Citraconate inhibits ACOD1 (IRG1) catalysis, reduces interferon responses and oxidative stress, and modulates inflammation and cell metabolism. Nat Metab (2022) 4:534–46. doi: 10.1038/s42255-022-00577-x PMC9170585 35655026

[200] Hooftman A O’Neill LAJ. The immunomodulatory potential of the metabolite itaconate. Trends Immunol (2019) 40:687–98. doi: 10.1016/j.it.2019.05.007 31178405

[201] Guillon A Brea-Diakite D Cezard A Wacquiez A Baranek T Bourgeais J. Host succinate inhibits influenza virus infection through succinylation and nuclear retention of the viral nucleoprotein. EMBO J (2022) 41:e108306. doi: 10.15252/embj.2021108306 35506364 PMC9194747

[202] Zhao J Chen J Wang C Liu Y Li M Li Y. Kynurenine-3-monooxygenase (KMO) broadly inhibits viral infections *via* triggering NMDAR/Ca2+ influx and CaMKII/ IRF3-mediated IFN-β production. PloS Pathog (2022) 18:e1010366. doi: 10.1371/journal.ppat.1010366 35235615 PMC8920235

[203] Liston A Masters SL. Homeostasis-altering molecular processes as mechanisms of inflammasome activation. Nat Rev Immunol (2017) 17:208–14. doi: 10.1038/nri.2016.151 28163301

[204] Hyrina A Burdette D Song Z Ramirez R Okesli-Armlovich A Vijayakumar A. Targeting lipid biosynthesis pathways for hepatitis b virus cure. PloS One (2022) 17:e0270273. doi: 10.1371/journal.pone.0270273 35925919 PMC9352027

[205] Gitto S Cicero AFG Loggi E Giovannini M Conti F Grandini E. Worsening of serum lipid profile after direct acting antiviral treatment. Ann Hepatol (2018) 17:64–75. doi: 10.5604/01.3001.0010.7536 29311405

[206] Meissner EG Lee YJ Osinusi A Sims Z Qin J Sturdevant D. Effect of sofosbuvir and ribavirin treatment on peripheral and hepatic lipid metabolism in chronic hepatitis c virus, genotype 1-infected patients. Hepatology (2015) 61:790–801. doi: 10.1002/hep.27424 25203718 PMC4340816

[207] Romero-Gómez M Rojas Á. Sofosbuvir modulates the intimate relationship between hepatitis c virus and lipids. Hepatology (2015) 61:744–7. doi: 10.1002/hep.27581 25345724

